# Comparative Efficacy of NAFLD Therapies and Biomarker Associations: A Meta-Analysis Based on Liver Fat Content

**DOI:** 10.1016/j.gastha.2024.100593

**Published:** 2024-11-29

**Authors:** Haoxiang Zhu, Ling Xu, Yinhua Lv, Juan Yang, Jihan Huang, Qingshan Zheng, Guang Ji, Lujin Li

**Affiliations:** 1 Center for Drug Clinical Research, Shanghai University of Traditional Chinese Medicine, Shanghai, China; 2 Institute of Digestive Disease, Longhua Hospital, Shanghai University of Traditional Chinese Medicine, Shanghai, China; 3 State Key Laboratory of Integration and Innovation of Classic Formula and Modern Chinese Medicine (Shanghai University of Traditional Chinese Medicine), Shanghai, China

**Keywords:** Nonalcoholic fatty liver, Nonalcoholic steatohepatitis, MRI-PDFF, MRS-PDFF

## Abstract

**Background and Aims:**

This study aims to conduct a comprehensive quantitative analysis of various nonalcoholic fatty liver disease (NAFLD) therapeutics, utilizing magnetic resonance (MR)-detected liver fat content (LFC) as the efficacy endpoint, and to identify biomarkers correlated with changes in LFC based on published literature.

**Methods:**

We performed a systematic search of public databases for placebo-controlled randomized trials on NAFLD up to September 29, 2023. A random-effects meta-analysis was employed to assess efficacy differences between drugs with various mechanisms and placebo. Initial Pearson correlation analysis explored the relationships between biomarkers and LFC. For biomarkers showing significant correlations with LFC, further modeling analysis was conducted to examine their relationship characteristics.

**Results:**

Our analysis included 36 studies with 3222 subjects and 33 investigational drugs, which were categorized into 6 mechanistic groups. Drugs such as fibroblast growth factor agonists, and those targeting adipocytes, inflammation, or fibrosis, showed greater efficacy in reducing LFC compared to Resmetirom, which has an efficacy of reducing LFC by 5.2%. From the 121 biomarkers analyzed, alanine aminotransferase and aspartate aminotransferase demonstrated moderate correlations with LFC; specifically, changes of −5.9 U/L in alanine aminotransferase or −3.3 U/L in aspartate aminotransferase were associated with an additional 1% reduction in LFC.

**Conclusion:**

The results of this study provide valuable insights for the clinical development of future NAFLD therapeutics, highlighting the efficacy of specific drug mechanisms and the potential of certain biomarkers as surrogate endpoints.

## Introduction

Nonalcoholic fatty liver disease (NAFLD) is a prevalent chronic liver condition,^1^ progressing from nonalcoholic fatty liver to nonalcoholic steatohepatitis (NASH), the latter characterized by fatty degeneration, lobular inflammation, and hepatocellular ballooning, with an estimated 40.76% risk of advancing to liver fibrosis.^2^ While lifestyle modifications and weight reduction are primary treatments, their challenges often lead patients to pharmacological options. In 2024, the Food and Drug Administration (FDA) approved Resmetirom as the first specific treatment for NASH.^3^ Additionally, drugs like vitamin E and pioglitazone, initially approved for other uses, are now included in clinical guidelines for NAFLD management due to their benefits in reducing liver inflammation and fibrosis.^4^

Numerous drugs, including fibroblast growth factor (FGF) agonists, Farnesoid X Receptor (FXR) agonists, Ketohexokinase inhibitors, peroxisome proliferator-activated receptor (PPAR) agonists, glucagon-like peptide-1 and antisense oligonucleotides are in development, with some showing positive results in Phase II trials.^5^^,^^6^ However, the lack of direct comparative studies among these classes makes their relative therapeutic potential unclear. Within the same drug class, such as FGF agonists, results vary significantly.^7^ Systematic quantitative analyses of current research are crucial to assess the therapeutic value of different drug classes and guide future medication development.

Liver biopsy is the gold standard for assessing the efficacy of NASH treatments and improvements in hepatitis and fibrosis,^8^ but its invasiveness leads to low patient compliance and complicates participant recruitment in clinical trials.^9^ Consequently, most Phase II trials now prefer noninvasive endpoints. For example, the FDA-approved drug Resmetirom used magnetic resonance imaging-proton density fat fraction (MRI-PDFF) as its primary endpoint in Phase II trials, and more precise measurements were achieved using magnetic resonance spectroscopy–based proton density fat fraction (MRS-PDFF) with magnetic resonance (MR) technology.^10^ Since NAFLD’s ultimate clinical goals are to halt or reverse inflammation and fibrosis—changes that manifest slowly—a reduction in liver fat content can directly reflect a drug’s efficacy in lipid metabolism and liver health improvement,^11^ also suggesting potential benefits on inflammation and fibrosis. Recognizing the value of noninvasive testing methods, the FDA has proactively embraced techniques like MRI and has conducted workshops to identify acceptable noninvasive testing approaches for NASH assessment.^12^ The European Medicines Agency, in its recent guidelines, has acknowledged MRI as an acceptable intermediate endpoint for future assessments.^13^ Thus, this study aims to conduct a comprehensive quantitative analysis of different NAFLD therapeutics using MR-detected liver fat content (LFC) as the efficacy endpoint.

Although MR technology improves patient compliance, its high costs and unsuitability for certain patients (eg, those with claustrophobia, larger body sizes, or metal implants) necessitate the identification of more accessible biomarkers as surrogate endpoints in NAFLD research.^14^ The exploration and validation of these biomarkers require diverse evidence from physiology, epidemiology, clinical trials, exposure–response relationships, and meta-analyses.^15^ Currently, no universally accepted biomarkers can precisely predict changes in LFC. The second research objective of this study is to explore biomarkers associated with changes in LFC based on data from published literature, aiming to conduct this exploration at the meta-analysis level.

## Methods

### Research Criteria and Eligibility

This study will adhere to the Preferred Reporting Items for Systematic Reviews and Meta-Analyses guidelines and its extensions for network meta-analyses (NMA.20, NMA.21, NMA.22).^16^^,^^17^ We will search PubMed and Embase for placebo-controlled randomized trials on NAFLD, restricting to English language publications from database inception to September 29, 2023. Details of the search strategies are available in the supplementary materials.

### Inclusion and Exclusion Criteria for Literature

Inclusion criteria include placebo-controlled, randomized, double-blind clinical trials that involve participants of all ages diagnosed with NAFLD and report outcomes of LFC measured by MR. Exclusion criteria include studies involving participants with diseases other than NAFLD, studies that selectively measure LFC, trials employing inconsistent methods for LFC measurement, and crossover trials lacking efficacy data from the first cycle.

Two reviewers (HX.Z and J.Y) will independently screen titles and abstracts to assess relevance. Full-text articles will be reviewed for definitive inclusion. Any disagreements will be resolved by a third reviewer (LJ.L).

### Data Extraction

Data for this study were extracted into Microsoft Excel (version 16.38) from each included study, capturing information on 1) publication basics such as first author, publication year, and clinical trial registration number; 2) trial specifics including the investigational drug, dosage, sample size, and trial duration; 3) participant characteristics like average age, gender distribution, baseline LFC, and nonalcoholic steatohepatitis scores; 4) outcome measures, namely changes in LFC and nonalcoholic steatohepatitis; and 5) biomarker details including names and values.

### Risk of Bias (RoB) Assessment

The quality of the included studies was assessed using the Cochrane Risk of Bias Tool (RoB2), which evaluates five domains: randomization process, deviations from intended interventions, missing outcome data, measurement of the outcome, and selection of the reported result. Each study’s RoB was categorized as “low,” “some concerns,” or “high.” Two independent reviewers (HX.Z and JS.Y) conducted the assessments, resolving any disagreements by consensus. Detailed results are available in the supplementary materials.

### Comparison of the Efficacy of Drugs with Different Mechanisms on LFC

This study used a reduction in LFC from baseline as the primary efficacy endpoint. A random-effects meta-analysis calculated the mean difference and 95% confidence interval (CI) between drug and placebo groups to assess efficacy differences across varying mechanisms of action. If a study lacked reported standard deviations, calculations followed Cochrane Handbook recommendations.^18^ Unavailable data prompted imputation using the median standard deviation from all included studies. Methodological details are in the supplementary materials.

Statistical heterogeneity among the studies was quantified using the I^2^ statistic. Values of I^2^ ≥ 50% indicated significant heterogeneity, prompting the use of a random-effects model for the meta-analysis. Conversely, if I^2^ < 50%, a fixed-effects model was employed. Potential publication bias was assessed using funnel plots.

The robustness and the potential sources of heterogeneity of the LFC results was evaluated through the following sensitivity analyses: 1) exclusion of studies with a high RoB; 2) exclusion of studies that used MRS-PDFF to measure LFC; and 3) exclusion of drugs that have been reported in only one study.

### Analysis of Biomarkers Related to LFC

The data analyzed in this study were derived from mean values reported in the literature. Based on the Central Limit Theorem, these means are generally assumed to follow a normal distribution. Consequently, Pearson correlation analysis was conducted to explore the relationship between biomarkers and LFC, with a Pearson coefficient above 0.4 indicating a correlation.^19^ To adjust for baseline and placebo effects, reductions in biomarkers and LFC were calculated separately for drug and placebo groups, followed by the computation of differences between these reductions. The change in biomarkers (ΔΔBio) was then used to predict the change in LFC (ΔΔLFC).

For biomarkers correlated with LFC, a predictive model was developed with ΔΔLFC as the dependent variable and ΔΔBio as the independent variable. Influencing factors such as age, gender ratio, weight, body mass index, baseline LFC and biomarkers, disease classification (nonalcoholic fatty liver/NASH), treatment duration, detection methods (MRI or MRS), publication year and center number (single/multicenter) were considered. This model quantifies the biomarker-LFC relationship and examines influencing factors. Detailed methods are available in the supplementary materials.

### Software

Literature management was conducted using Endnote X8. Statistical analyses and result visualization were performed using R (version 4.0.2) and R Studio (version 1.2.5033), with meta-analyses implemented through the “meta” package. Modeling was executed using NONMEM (Version 7.4; Icon Inc, PA, USA), employing the First-Order Conditional Estimation method for estimating model parameters.

## Results

### Characteristics of Included Studies

A total of 209 articles were retrieved, with 36 articles containing 59 trial groups meeting the inclusion criteria. The selection process is illustrated in Figure S3, and a list of included articles is in the supplementary. These articles encompassed 3222 participants: 2050 in drug groups and 1172 in placebo groups. Participant ages ranged from 11 to 64 years, with a median of 52. The percentage of male participants ranged from 17% to 90.9%, with a median of 50%; and body weight ranged from 66 to 131.1 kg, with a median of 94.2 kg. Baseline LFC varied from 6.8% to 38.8%, with a median of 19.1%. The follow-up period was 12–52 weeks, with a median of 21 weeks. MRI-PDFF was the primary detection method, used in 78% of cases; 59.3% of trials occurred in North America and 72.9% were multicenter. Detailed characteristics of the participants and trials are shown in Table 1 and Table A9; further details are provided in the supplementary.

**Table 1:** Characteristics of Trials and Baseline Characteristics of Subjects Included in the Review, Median (Minimum, Maximum)

Characteristic	FGF agonist (arm = 15)	CF (arm = 4)	FXR agonist (arm = 6)	LCM (arm = 13)	Others (arm = 16)	PPAR agonist (arm = 5)	Overall (arm = 59)
Sample size	30 (6, 43)	51 (21, 80)	54 (19, 56)	35 (13.4, 90)	22.5 (6, 79)	27 (25, 58)	30 (6, 90)
Trial period	24 (12, 26)	24 (21, 26)	12 (12, 24)	16 (12, 52)	18.4 (12, 52)	16 (16, 24)	21 (12, 52)
Age, y	52.4 (49.5, 56.4)	41.5 (40.3, 42.6)	53.2 (51, 58)	53.7 (11, 64)	51.2 (39.1, 61.5)	49 (43.9, 53.2)	52 (11, 64)
Male, %	41 (17, 89)	78.5 (71, 86)	39 (26.3, 47.3)	52.4 (29, 88)	63.2 (45, 90.9)	52 (27, 55.6)	50 (17, 90.9)
White, %	92 (82, 100)	NA	80.2 (75, 88.7)	71.9 (44, 100)	89 (83, 95)	84.3 (0, 88.9)	86.5 (0, 100)
Weight (kg)	101.5 (87.1, 131.1)	81.5 (80.8, 82.1)	94.9 (92.4, 101)	91.9 (66, 101.7)	92.8 (78.1, 103.2)	80 (-)	94.2 (66, 131.1)
Body mass index (kg/m^2^)	36.8 (32.7, 38.8)	30.7 (33.52, 28.4)	34.2 (32.7, 37)	32.3 (28.9, 34.4)	32.9 (24.3, 38.8)	28.95 (28.4, 29.5)	32.9 (24.3, 38.8)
Liver fat content %	19.4 (17.4, 22.4)	26 (19.96, 38.8)	18.8 (14.9, 22)	15.3 (9.91, 30.2)	18.6 (6.8, 26.9)	20.2 (18.7, 23.9)	19.1 (6.8, 38.8)

CF, drugs acting on cell death, inflammation, and fibrosis; LCM, drugs acting on glucose and lipid metabolism.

### Categories of Drugs Included in the Analysis

**Figure 1:**
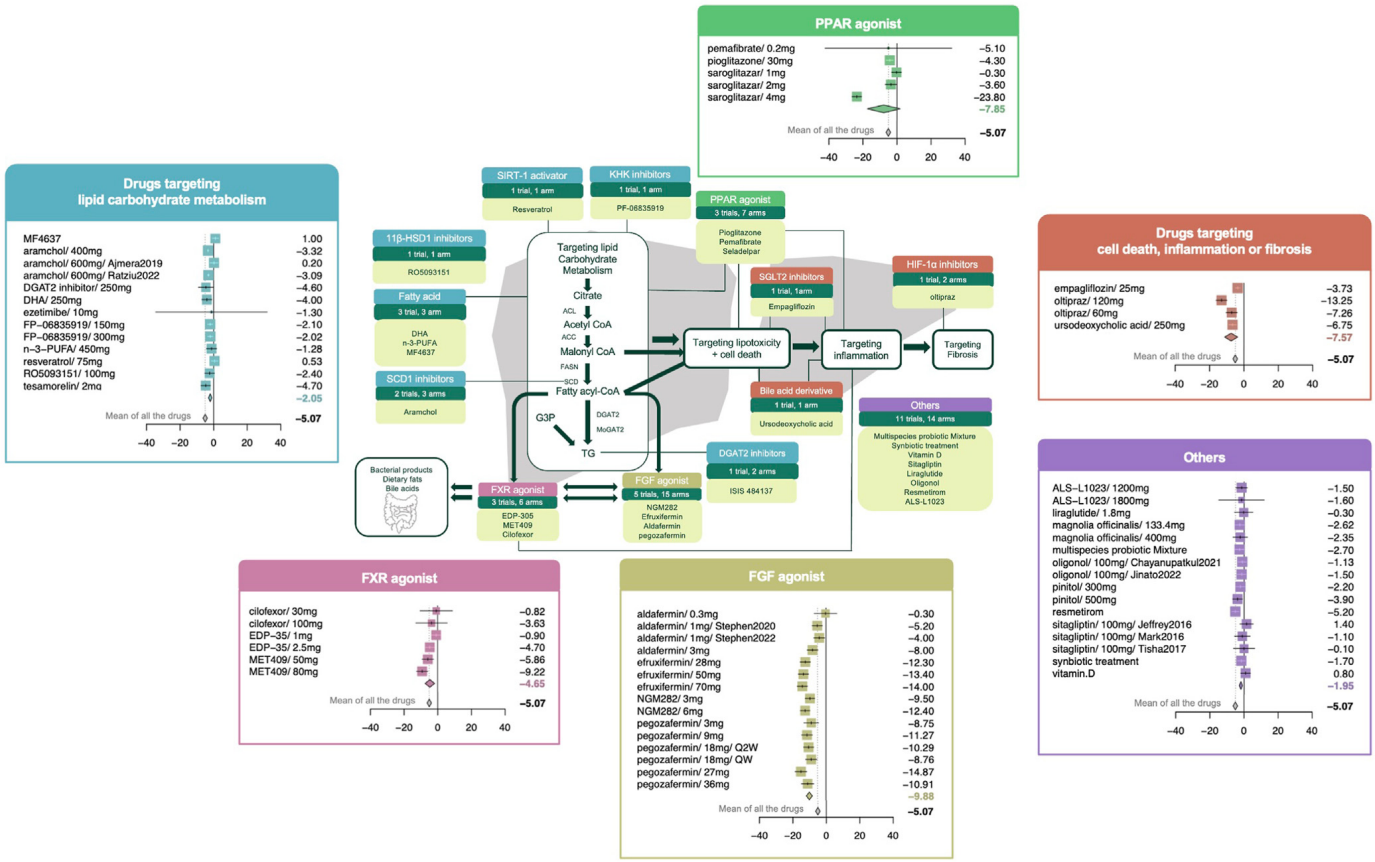
Forest plot of meta-analysis on the reduction of LFC by NAFLD treatment drugs with different mechanisms of action.^20^ Drugs were categorized based on their mechanisms of action, and each category was represented by a distinct color in the forest plots: blue for drugs targeting glucose and lipid metabolism, red for drugs impacting cell death, inflammation, or fibrosis, yellow for FGF agonists, pink for FXR agonists, green for PPAR agonists, and purple for drugs belonging to other categories. The forest plots, contained within boxes of corresponding colors, display the meta-analysis results for each drug category. The average efficacy of all drugs is represented by gray diamond points in the plots.

This study analyzed 33 investigational drugs, categorized into 6 groups based on their mechanisms of action (Figure 1).

1) Carbohydrate and lipid metabolism agents: includes aramchol, docosahexaenoic acid, ezetimibe, FP-06835919, ISIS 484137, MF4637, n-3-polyunsaturated fatty acid, resveratrol, RO5093151, and tesamorelin, totaling 13 groups;
2) Adipocyte, inflammation, or fibrosis targeting drugs: empagliflozin, oltipraz, and ursodeoxycholic acid, comprising 4 trial groups;
3) FXR agonists: Ilofexor, EDP-35, and MET409, with 6 trial groups;
4) FGF agonists: aldafermin, efruxifermin, NGM282, and pegozafermin, encompassing 15 trial groups.
5) PPAR Agonists: pemafibrate, pioglitazone, and saroglitazar, totaling 5 trial groups.
6) Other drugs: includes liraglutide, magnolia officinalis, a multispecies probiotic mixture, oligonol, pinitol, resmetirom, sitagliptin, synbiotic treatment, and vitamin D, comprising 16 trial groups.

### Comparative Efficacy in Reducing LFC

**Figure 2:**
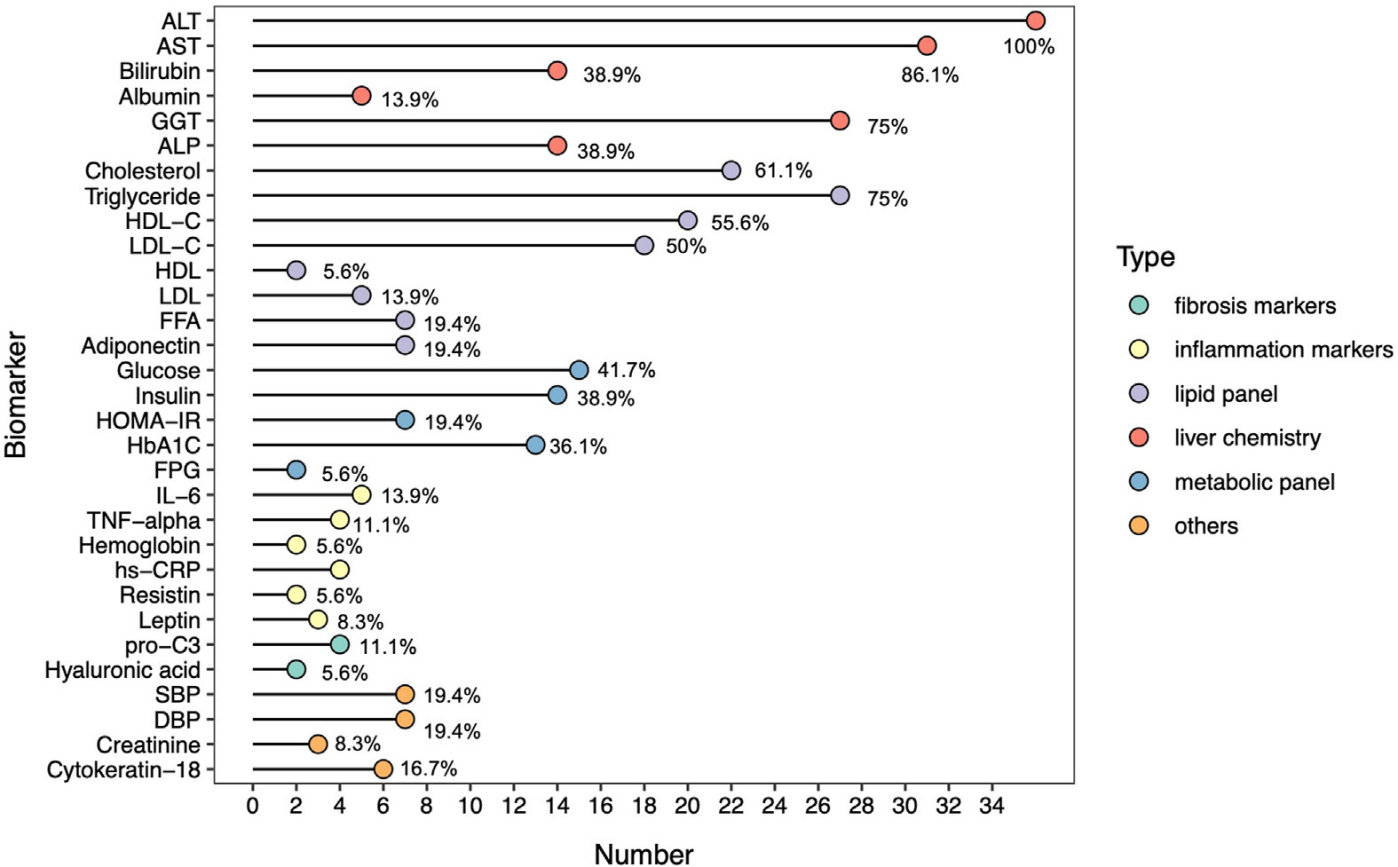
Distribution of the number and proportion of biomarkers reported in the literature. Abbreviation: GGT, Gamma-glutamyl transferase; ALP, alkaline phosphatase, HDL-C, high-density lipoprotein cholesterol; LDL-C, low-density lipoprotein cholesterol; HDL, high-density lipoprotein; LDL, low-density lipoprotein; FFA, free fatty acids; HbA1c, hemoglobin A1c; FPG, fasting plasma glucose; IL-6, interleukin-6; TNF-alpha, tumor necrosis factor-alpha; hs-CRP, high-sensitivity C-reactive protein; pro-C3 Procollagen type III N-terminal propeptide; SBP, systolic blood pressure, DBP, diastolic blood pressure.

In a comparison with the placebo group, all drug groups collectively achieved a significant mean LFC change of −5.07% (95% CI: −6.39 to −3.74) (see Figure 2 and Figure A10.1). Among the drug categories, FGF agonists were the most effective, with an average LFC change of −9.88% (95% CI: −11.67 to −8.1), despite significant heterogeneity (I^2^ = 75%, τ^2^ = 9.2751, *P* < .01). Pegozafermin (27 mg) led with a −14.87% change (95% CI: −18.41 to −11.33), followed by efruxifermin (70 mg) and NGM282 (6 mg) with −14.00% (95% CI: −17.1 to −10.9) and −12.40% (95% CI: −15.27 to −9.53), respectively. Aldafermin (0.3 mg) was least effective, changing LFC by only −0.30% (95% CI: −7.06 to 6.46).

Drugs targeting adipocytes, inflammation, or fibrosis significantly changed LFC by an average of −7.57% (95% CI: −11.44 to −3.7), also with high heterogeneity (I^2^ = 90%, τ^2^ = 13.7665, *P* < .01). Individual changes were −13.25% by oltipraz (120 mg) (95% CI: −16.54 to −9.95), −7.26% by oltipraz (60 mg) (95% CI: −10.58 to −3.93), and −6.75% by ursodeoxycholic acid (95% CI: −8.98 to −4.52), −3.73% by empagliflozin (95% CI: −5.2 to −2.26).

Other drug categories such as those acting on carbohydrate and lipid metabolism, and FXR agonists showed relative lower but still significant efficacy compared to placebo, with change of −2.05% (95% CI: −3.09 to −1.01), −4.65% (95% CI: −7.52 to −1.77), and −1.95% (95% CI: −2.91 to −1), respectively. Within these, tesamorelin, MET409 (80 mg), and resmetirom were top performers, achieving reductions of −4.70% (95% CI: −7.51 to −1.89), −9.22% (95% CI: −12.68 to −5.76), and −5.20% (95% CI: −7.35 to −3.05), respectively.

PPAR agonists altered LFC by an average of −7.85% (95% CI: −17.62 to 1.92), which did not significantly differ from placebo. This was primarily due to the saroglitazar 4-mg group exhibiting a significantly greater reduction in LFC compared to other drug groups. The large variability across trials contributed to the wide 95% CI. Consequently, the results suggesting that PPAR agonists reduce LFC are not robust.

Due to limited data and substantial heterogeneity in efficacy values for certain categories of drugs, funnel plots are ineffective in determining the presence of publication bias. (Figure A15)

Sensitivity analyses demonstrated that excluding studies with high bias risk, those employing MRS-PDFF for LFC measurement and drugs that have been reported in only one study, did not significantly alter the LFC outcomes across different drug classes, indicating the robustness of the LFC conclusions drawn in this study. Additionally, after excluding these potentially heterogeneous studies, the I^2^ values did not significantly decrease, suggesting that these factors are not the main sources of heterogeneity. Instead, heterogeneity primarily stems from the differences in efficacy among various types of drugs within the same category (Table A11, Figure A11.1–11.3).

### Biomarker Correlation Analysis with LFC

**Figure 3:**
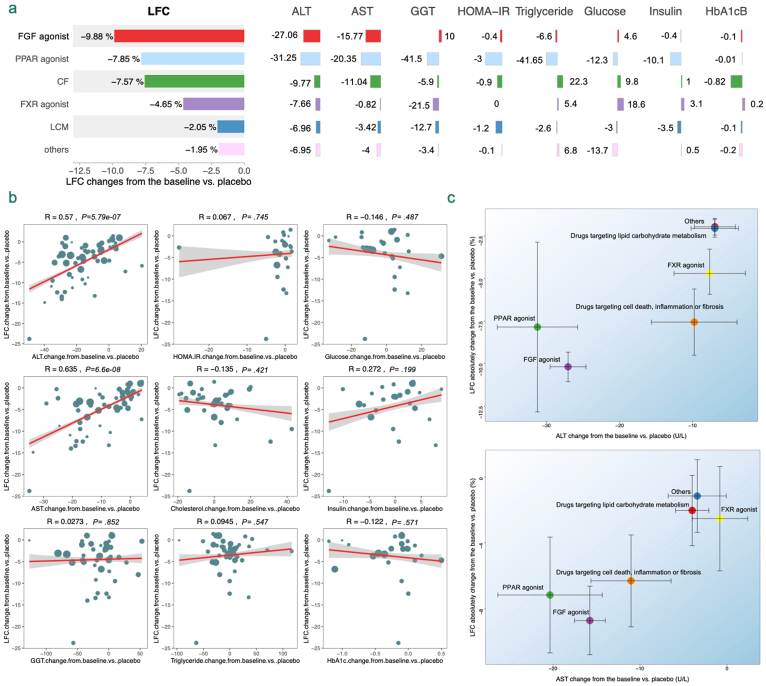
Exploratory analysis of the correlation between biomarkers and LFC. A) Distribution of meta-analysis results for changes in LFC and biomarkers from baseline relative to placebo across different drug categories; B) Scatter plot of correlations between different biomarkers and changes in LFC. Scatter points representing the changes in LFC and corresponding changes in various biomarkers, adjusted for baseline and placebo effects. The red line indicates the trend of correlation between changes in LFC and biomarker values, with the shaded area representing the 95% CI of the fitted line. The correlation coefficient from Pearson’s correlation analysis is denoted by R; C) Scatter plot of the correlation between LFC and ALT/AST by drug category. Scatter points representing the mean changes in LFC and corresponding changes in ALT or AST for each drug category, adjusted for baseline and placebo effects. The black lines indicate the standard error of the means.

This study analyzed 36 articles, identifying 121 biomarkers, with 31 reported in 2 or more articles (Figure 2). This study analyzed the correlation between changes in 9 key biomarkers and ΔΔLFC. Moderate correlations were observed between alanine aminotransferase (ALT) and aspartate aminotransferase (AST) with ΔΔLFC, with correlation coefficients (r) of 0.57 and 0.635, respectively. Conversely, Insulin showed weaker correlations with ΔΔLFC, with coefficients of 0.272. Other biomarkers including the Homeostatic Model Assessment of Insulin Resistance (HOMA-IR), insulin, gamma-glutamyl transferase, triglyceride, and hemoglobin A1c showed no significant correlations (Figure 3).

Given these correlations, the study developed a predictive linear model for ΔΔLFC using ΔΔAST or ΔΔALT, supported by exploratory data indicating a linear relationship (Figure 3). The modeling process and covariate analysis details are provided in the supplementary materials, revealing no significant covariate effects on model parameters. The final model expression is as follows:

Equation 1 ΔΔLFC=0.17×ΔΔALT−1.53

Equation 2 ΔΔLFC=0.3×ΔΔAST−1.51

**Figure 4:**
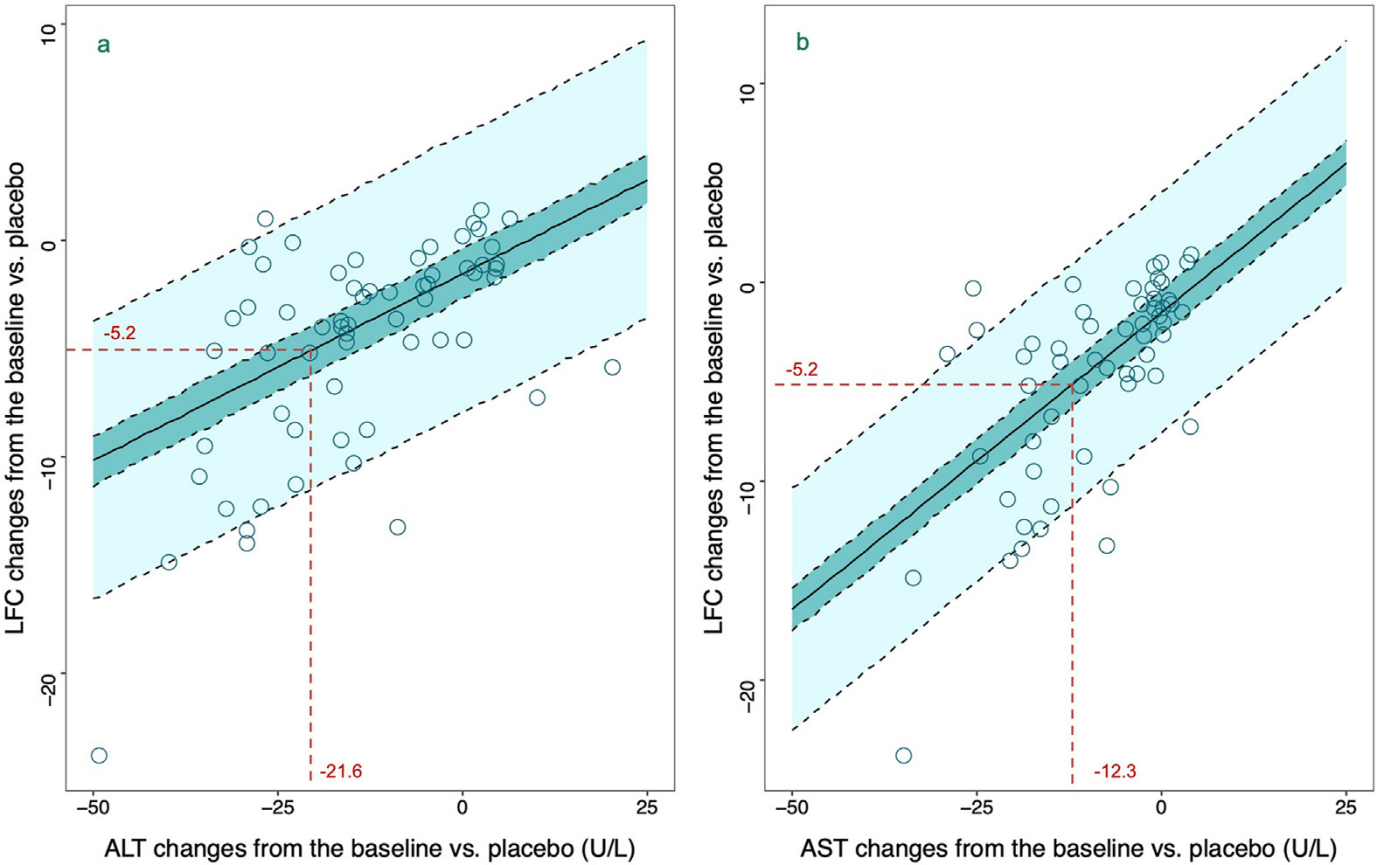
Visual predictive check plots for ALT predicting LFC (A) and AST predicting LFC models (B). Scatter points representing the adjusted mean changes in LFC and corresponding changes in ALT or AST from each included study, after adjustments for baseline and placebo effects. The light shaded area represents the 95% CI of the model predictions, while the dark shaded area indicates the 95% CI of the median predictions of the model.

Equations 1 and 2 show that a change of −5.9 U/L in ALT or −3.3 U/L in AST corresponds to a 1% decrease in LFC. Table 2 shows the parameters of the model and the bootstrap results. Bootstrap analysis results align with model parameters, indicating stability. Visual predictive check results confirm that observed data fall within the 95% prediction interval of model simulations, illustrating robust predictive performance (Figure 4).

**Table 2:** Estimates of Model Parameters for ALT and AST Predicting LFC and Bootstrap Results

	ALT-LFC model		AST-LFC model	
	Estimate (RSE%)	Bootstrap (95% CI)	Estimate (RSE%)	Bootstrap (95% CI)
Fixed effect parameter				
θ (α)	0.17 (31.6)	0.17 (0.07−0.27)	0.3 (24.9)	0.3 (0.16−0.45)
θ (b)	−1.53 (38.4)	−1.55 (−2.67 to −0.61)	−1.51 (31.5)	−1.5 (−2.65 to −0.70)
Residual error				
ε	21.66 (11.8)	20.7 (15.8−26.0)	20.7 (10.7)	20.1 (15.5−24.5)

RSE, recurrent spontaneous epistaxis.

## Discussion

Liver biopsy is currently the gold standard for diagnosing NAFLD in clinical trials. However, due to the challenges and difficulties associated with obtaining biopsies and their high clinical implementation costs, an increasing number of Phase II clinical trials are shifting focus to explore noninvasive biomarkers instead. MRI, validated as an accurate and noninvasive method, closely correlates with liver histology for estimating LFC and differentiating levels of hepatic steatosis. Studies indicate that MRI correlates well with liver histology in assessing hepatic steatosis and can distinguish between varying degrees of fatty liver degeneration. Ismail’s research further confirms a strong correlation between MR-estimated LFC and liver biopsy results.^21^ These findings suggest MR is a reliable and accurate tool for evaluating LFC and detecting NAFLD, potentially replacing liver biopsy and avoiding its associated risks such as bleeding and infection. This study used MR-detected LFC to evaluate the efficacy of drugs with various mechanisms in treating NAFLD. Results showed significant LFC reductions across all drug categories compared to placebo, with FGF agonists, particularly FGF-19 and FGF-21, demonstrating the most substantial effects. FGF-19, mainly from the intestine, regulates bile acid synthesis and lipid metabolism, with agonists like NGM282 and aldafermin showing dose-responsive reductions in liver fat and fibrosis.^22^ FGF-21, produced by the liver, enhances lipid and glucose metabolism and reduces inflammation.^23^ FGF-21 agonists such as efruxifermin and pegozafermin have also shown promising results in reducing liver fat and improving NASH markers, with pegozafermin at 27 mg achieving a notable ΔΔLFC of 14.87%. Despite their potential, the long-term effects and safety of FGF agonists require further investigation, with attention to possible adverse effects like nausea and diarrhea.^24^

In March 2024, resmetirom was approved by the FDA as the first and only drug for treating NASH. It is a selective thyroid hormone receptor β agonist that enhances lipid metabolism and fatty acid oxidation in the liver, leading to reduced low-density lipoprotein and triglyceride levels.^25^ In phase II trials, resmetirom achieved a ΔΔLFC of −5.2%.^10^ While the clinical significance of this reduction remains unclear, it establishes a benchmark for evaluating other NAFLD treatments. Our study found that the efficacy of FGF agonists, PPAR inhibitors, and drugs targeting adipocytes, inflammation, or fibrosis exceeded that of resmetirom, highlighting their potential in future drug development. In contrast, drugs affecting glycolipid metabolism, and other classes showed lower efficacy in reducing LFC, suggesting a cautious approach to their further development. This study preliminarily explored the potential of different categories of investigational drugs to reduce LFC, thereby indicating their possible efficacy in treating NAFLD. However, since most of these drugs are still in the development phase and the studies published so far have involved small sample sizes, the results may be subject to sampling errors. Consequently, these findings will need to be validated in future large-scale clinical trials.

Given the high cost of MRI, this study analyzed biomarkers associated with LFC, identifying hepatic enzymes ALT and AST, and lipid markers cholesterol and triglycerides. Employing ΔΔBio and ΔΔLFC values for correlation analysis minimized confounding factors, revealing a moderate correlation between ALT, AST, and LFC. The study also demonstrated that drugs with various mechanisms not only reduced LFC but also significantly decreased ALT and AST levels, proportionate to LFC reduction. Although ALT and AST, indicators of liver damage, are influenced by conditions like alcoholic liver disease, viral hepatitis, and drug toxicity,^26^^,^^27^ their reduction still indirectly evidences drug efficacy in lowering LFC at a population level. We observed that the phase III clinical data of Resmetirom has been published,^28^ and we compared its results with our model (Figure A16). The findings indicate that the data fall within the predictive interval of the model and is close to the 90% CIs of the median. These results are consistent with the findings of our study. This study provides clinical reference value, showing that a ΔΔLFC of −5.2% corresponds with reductions of −21.6 U/L in ALT and −12.3 U/L in AST, offering valuable insights for NAFLD treatment development.

HOMA-IR is an important index for assessing insulin resistance, which is closely linked to NAFLD development.^29^^,^^30^ Despite its potential as a surrogate marker for LFC and recommendations in some clinical guidelines based on individual or animal studies,^31^ our analysis of population-based data does not support HOMA-IR as a reliable surrogate. This finding is crucial for researchers considering the use of HOMA-IR as an alternative endpoint in studies. Future research in the field of NAFLD should place greater emphasis on developing and validating more reliable biomarkers. By focusing on the investigation and validation of novel biomarkers with strong predictive capabilities, researchers can gain a deeper understanding of the pathophysiology of NAFLD and potentially identify new therapeutic targets, thereby facilitating the development of more effective treatment methods.

This study included a broad dataset by not limiting the magnetic resonance imaging techniques used, covering both MRI-PDFF and MRS-PDFF. Among various noninvasive imaging modalities used for the hepatic fat assessment, MRI and MRS are the most accurate methods, as they directly measure the proton signals in water and fat. Several MRI-based methods (e.g., Dixon technique) have been introduced for the measurement of PDFF and are more widely available than MRS.^32^ The study’s findings indicate that the choice between MRI and MRS methods did not significantly influence the outcomes (Figure A11.2).

This study has several limitations. First, this study evaluated the efficacy of pharmacotherapy for NAFLD solely based on LFC, without considering comprehensive assessments of inflammation and fibrosis. Future investigations will incorporate these parameters as relevant data become available. Second, LFC measurements were only taken at baseline and endpoint, preventing the construction of time-course models for drug efficacy onset. The trial durations varied from 12 to 52 weeks, introducing potential heterogeneity; however, exploratory analyses found no significant impacts of duration on drug efficacy (Figures A13). Third, the limited number of trials per drug hindered the development of dose–response models, although meta-analysis suggested a dose–response relationship for some drugs. Future research aims to establish these models to identify the minimum effective doses. Additionally, only one study provided both MRI and biopsy results, limiting correlation analysis between these methods, despite known close relationships. The study relied on aggregated literature data, often lacking details like ethnicity and diabetes status, which could affect outcomes. Furthermore, 16 included studies had a high RoB; however, Sensitivity analysis excluding these studies showed consistent results, affirming the robustness of the conclusions. Lastly, the inclusion of only English-language literature may introduce publication bias.

## Conclusion

This study used MR-measured LFC as the primary endpoint to evaluate various drug mechanisms under development for NAFLD treatment. Results showed that FGF agonists and drugs targeting adipocytes, inflammation, or fibrosis were more effective in reducing LFC than Resmetirom, the only FDA-approved NASH treatment. This underscores their potential clinical importance and future development prospects. The study also reviewed NAFLD-related biomarkers, finding moderate correlations between LFC and the liver enzymes ALT and AST, despite their lack of specificity as direct LFC markers. However, population-level changes in ALT and AST can support the assessment of drug efficacy in clinical trials, complementing LFC measurements. These findings provide valuable insights for the clinical development of future NAFLD therapies.
