# Supplementary material for: Comparative Efficacy of NAFLD Therapies and Biomarker Associations: A Meta-Analysis Based on Liver Fat Content
Source: Gastro Hep Adv. 2024 Nov 29;4(4):100593. doi: 10.1016/j.gastha.2024.100593 (PMC11869496; doi:10.1016/j.gastha.2024.100593)
Supplement: Supplementary Material [file mmc1.docx]

**Supplementary**

[1. PubMed search query 2](#_Toc178866206)

[2. Embase search query 3](#_Toc178866207)

[3. Literature inclusion and exclusion process 4](#_Toc178866208)

[4. Detailed information on biomarkers reported in the literature 5](#_Toc178866209)

[5. Baseline characteristics of all included literatures 8](#_Toc178866210)

[6. List of the included references 11](#_Toc178866211)

[7. Details of modeling analysis 16](#_Toc178866212)

[Covariate analysis 16](#_Toc178866213)

[Model evaluation 16](#_Toc178866214)

[8. Missing SD data imputation method 17](#_Toc178866215)

[9. Risk of bias 18](#_Toc178866216)

[10. Meta-analysis results for ALT, AST, and LFC 19](#_Toc178866217)

[11. Results of Subgroup analysis 22](#_Toc178866218)

[12. Time-course characteristics of △△LFC 25](#_Toc178866219)

[13. Baseline characteristic of the included literature 26](#_Toc178866220)

[14. List of excluded literatures after full-text reading 27](#_Toc178866221)

[15. Publication bias analysis 34](#_Toc178866222)

[16. Comparison of observed data and model predictions in the Phase III clinical trial of Resmetirom 35](#_Toc178866223)

| **Search**  **number** | **Query** | **Results** |
| --- | --- | --- |
| 1 | NAFLD[Title/Abstract] | 25,515 |
| 2 | non-alcoholic fatty liver disease[Title/Abstract] | 16,697 |
| 3 | NASH[Title/Abstract] | 13,537 |
| 4 | nonalcoholic steatohepatitis[Title/Abstract] | 7,993 |
| 5 | placebo[Title/Abstract] | 248,751 |
| 6 | "liver biopsy" | 21,907 |
| 7 | "magnetic resonance" | 883,787 |
| 8 | MR | 282,781 |
| 9 | MRI | 754,421 |
| 10 | (((NAFLD[Title/Abstract]) OR (non-alcoholic fatty liver disease[Title/Abstract])) OR (NASH[Title/Abstract])) OR (nonalcoholic steatohepatitis[Title/Abstract]) | 38,232 |
| 11 | (("magnetic resonance") OR (MR)) OR (MRI) | 1,130,050 |
| 12 | (((((NAFLD[Title/Abstract]) OR (non-alcoholic fatty liver disease[Title/Abstract])) OR (NASH[Title/Abstract])) OR (nonalcoholic steatohepatitis[Title/Abstract])) AND (placebo[Title/Abstract])) AND ((("magnetic resonance") OR (MR)) OR (MRI)) | 131 |
| 13 | (((((NAFLD[Title/Abstract]) OR (non-alcoholic fatty liver disease[Title/Abstract])) OR (NASH[Title/Abstract])) OR (nonalcoholic steatohepatitis[Title/Abstract])) AND (placebo[Title/Abstract])) AND ((("magnetic resonance") OR (MR)) OR (MRI)) | 99 |
| 14 | (((((NAFLD[Title/Abstract]) OR (non-alcoholic fatty liver disease[Title/Abstract])) OR (NASH[Title/Abstract])) OR (nonalcoholic steatohepatitis[Title/Abstract])) AND (placebo[Title/Abstract])) AND ((("magnetic resonance") OR (MR)) OR (MRI)) | 99 |

## 1. PubMed search query

## 2. Embase search query

| **No.** | **Query** | **Results** |
| --- | --- | --- |
| 1 | 'non-alcoholic fatty liver disease' | 28123 |
| 2 | nafld | 44221 |
| 3 | 'nonalcoholic steatohepatitis' | 20214 |
| 4 | nash | 41325 |
| 5 | mr | 245490 |
| 6 | mri | 622754 |
| 7 | 'magnetic resonance' | 1704436 |
| 8 | placebo | 533898 |
| 9 | #1 OR #2 OR #3 OR #4 | 83144 |
| 10 | #5 OR #6 OR #7 | 1831107 |
| 11 | #8 AND #9 AND #10 | 542 |
| 12 | #8 AND #9 AND #10 AND ([randomized controlled trial]/lim OR 'controlled clinical trial'/de) | 296 |
| 13 | #12 AND 'Article'/it | 110 |

## 3. Literature inclusion and exclusion process


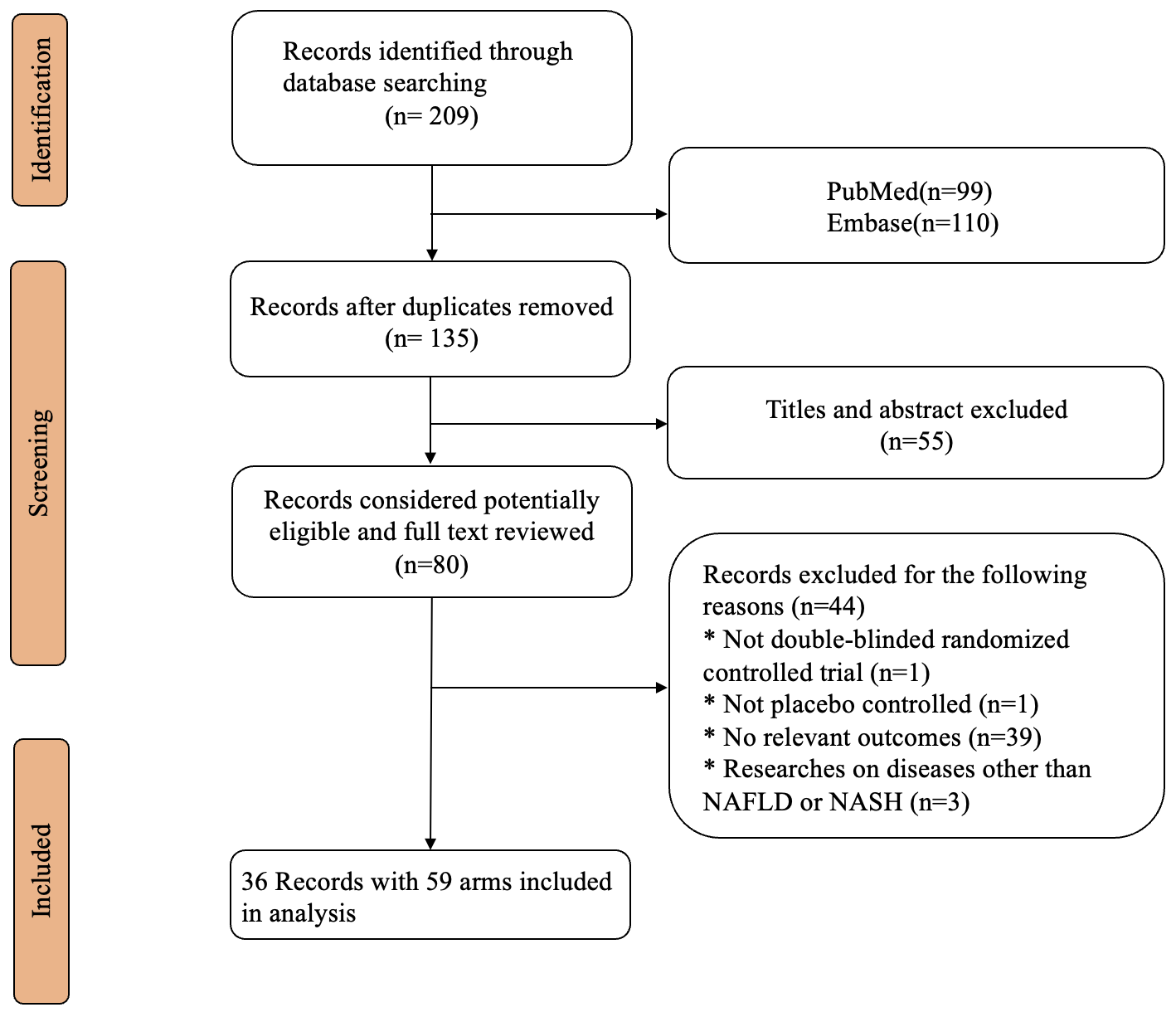


FigureS3. Literature inclusion and exclusion process

## 4. Detailed information on biomarkers reported in the literature

Table S4：Summary of biomarkers reported in the literature

| **Author** | **Publish year** | **Reported biomarkers** |
| --- | --- | --- |
| sang Bong Ahn | 2019 | ALT, AST, Cholesterol, Triglyceride, HDL-C, Glucose, insulin, HOMA-IR, IL-6, TNF-a, LPS |
| Veeral H. Ajmera | 2019 | ALT, AST, ALP, Bilirubin (T/D), Albumin, Triglycerides, cholesterol, LDL, FFA, Glucose, Insulin, HOMA-IR, Hemoglobin AIC, CD4 count |
| Ilaria Barchetta | 2016 | 25(OH)D, SBP, DBP, Cholesterol, HDL-C, LDL-C, Triglycerides, FBG, HbA1c, AST, ALT, GGT, CK8-M30, P3NP, FFAs, FBI, FLI, HOMA-IR, HOMA-BETA%, QUICKI, ADIPO-IR, CRP, Adiponectin |
| Eleonora Scorletti | 2020 | SBP, DBP, Glucose, Insulin, HbA1c, TC, TG, HDL-C, LDL-C, ALT, AST, GGT, HA, PIIINP, TIMP-1, LPS, Bifidobacterium spp, Acetic Acid, Propionic Acid, Butyric Acid |
| Jeffrey Cui | 2016 | ALT, AST, Glucose, Insulin, HbA1C, Triglycerides, Cholesterol, LDL, FFA, ALK Phos, GGT, Bilirubin (T/D), Abumin, protime, HOMA-IR |
| Samer Gawrieh | 2019 | ALT, AST, ALP, GGT, Triglycerides, LDL, VLDL, HDL, Total Cholesterol, HbA1c, FPG, HOMA-IR, Insulin, Creatinine, Adiponectin, Cytokeratin-18 |
| Stephen A Harrison, Guy Neff | 2020 | ALT, AST, ALP, GGT, bilirubin, Pro-C3, Hyaluronic acid, PIIINP, TIMP-1, Glucose, Insulin, HOMA-IR, HbA1c, cholesrerol, HDL-C, LDL-C, Triglyerides |
| Stephen A Harrison | 2018 | ALT, AST, ALP, GGT, bilirubin, Cholesterol, HDL-C, LDL-C, Triglycerides, C4, Hemoglobin, Hematocrit, Mean corpuscular volume, White blood cell count, Platelet count, Bicarbonate, Calcium, Phosphate, Uric acid, Albumin, INR, Glucose, HbA1c, Insulin, HOMA-IR |
| Stephen A Harrison | 2021 | ALT, AST, GGT, ALP, Urate, pro-C3 |
| Jee‐Fu Huang | 2021 | ALT, AST, hs-CRP, HOMA-IR, FPG, HbA1c, EOT |
| Jae Yoon Jeong | 2017 | AST, ALT, TC, TG, HDL-C, LDL-C, VLDL-C, FFA, HOMA-IR |
| Tisha R Joy | 2016 | HbA1C, HOMA-IR, Triglycerides, HDL-C, LDL-C, Free fatty acids, Platelet aggregation, AST, ALT, Aik phos, GGT, Adiponectin, Adipsin, Visfatin, Leptin, Resistin, TNF-a, IL-6 |
| Konstantinos Kantartzis | 2018 | HbA1C, HOMA-IR, ISI, Glucose, cMIT, VO2max, SBP, DBP, TC, HDL-C, LDL-C, TG, AST, ALT, AUC of FFA, AST, GGT, hsCRP, IL-6, Fetuin-A, SHBG, CK-18 M30 |
| W. Kim | 2017 | Insulin, Glucose, HOMA-IR, ALT, AST, GGT, Total cholesterol, LDL-C, HDL-C, VLDL-C, Triglyceride, Free fatty acid, TNF-a, IL-6, Adiponectin, Resistin, Leptin |
| Eunok Lee | 2019 | ALT, AST, GGT, Postprandial Triglycerides |
| Rohit Loomba | 2015 | ALT, AST, Glucose, Insulin, Hgb A1C, Triglycerides, Cholesterol, LDL, FFA, ALK Phos, GGT, Bilirubin (T/D), HOMA-IR |
| Rohit Loomba | 2020 | Triglycerides, Cholesterol, ALT, AST, GGT |
| Atsushi Nakajima | 2021 | ALT, AST, GGT, ALP, Total bilirubin, Platelets, Total cholesterol, CK-18 M30, M2BPGi,Hyaluronic acid, 7S domain of type IV collagen, ELF, eGFR, Creatinine |
| L. Pacifico | 2015 | Systolic blood pressure, Diastolic blood pressure, ALT, cholesterol, HDL-C, Triglycerides, Glucose, Insulin, HOMA-IR, WBISI, HSCRP, DHA |
| V. Ratziu | 2022 | ALT, AST, ALP, GGT, Bilirubin, TG, TC, LDL-C, HDL-C, Glucose, HbA1c, Fibrosis, NFS, FLI |
| Vlad Ratziu | 2022 | ALT, AST, MRE, Procollagen-C3, Hyaluronic aid, PIIINP, TIMP-1, AST, GGT, Billrubin, ALP, Triglycerides, Cholesterol, Apolipoprotein, Fasting glucose, Fasting insulin, HbA1c, Adiponectin, Fibrinogen, HS-CRP, IL-6,IL-1beta, TNF-a, Haptoglobin, Alpha2, macroglobulin, Glucagon-like peptide-1, Cytokeratin-18 |
| Václav Šmíd | 2022 | ALT, AST, GGT, HbA1c, TG, TC, LDL-C, HDL-C |
| Mark M. Smits | 2016 | Glucose, Insulin, HbA1c, AST, ALT, GGT, ALP, Albumin, Bilirubin, L-FABP |
| Takara L Stanley | 2019 | ALT, GGT, IGF-1, TG, HDL-C, LDL-C, CRP, Adiponectin, glucose, HbA1c, CD4, CD8 |
| Norbert Stefan | 2014 | TC, HDL-C, LDL-C, TG, SBP, DBP, ALT, AST, GGT |
| Derek Tobin | 2018 | SBP, DBP, Heart rate, Fasting glucose, Fasting insulin, HbA1c, Triglycerides, BUN, Creatinine, TSH μIU, Hs-CPR, Albumin, ALT, AST, ALP, GGT, Bilirubin, RBC fatty acid content |
| Maneerat Chayanupatkul | 2022 | ALT, FBS, HOMA-IR, TG, TC, LDL-C, HDL-C, bilirubin, MDA, Leptin, CK-18 |
| Sahar H. Elhini | 2022 | SBP, DBP, AST, ALT, ALP, GGT, FG, PPG, HbA1c, HOMA-IR, HOMA-B, Insulin, eGFR |
| Stephen A Harrison | 2022 | ALT, AST, ALP, GGT, bilirubin, TC, HDL-C, LDL-C, Triglycerides |
| Thananya Jinato | 2022 | ALT, AST, FBS, TC, TG, HDL, LDL, HOMA-IR |
| Rohit Loomba | 2022 | ALT, AST, PRO-C3, TG, LDL-C, HDL-C |
| Aditi R. Saxena | 2023 | ALT, AST, ALP, GGT, HbA1c, glucose, insulin, HOMA-IR |
| Keyur | 2020 | ALT, AST, GGT, ALP, FGF19, C4, Bille acids |
| Stephen A Harrison | 2019 | ALT, AST, GGT, bilirubin, ALP, LDL-C, HDL-C, Lipoprotein, Apolipoprotein B, Triglycerides, Apolipoprotein CIII, N-terminal type III collagen propeptide, Cytokeratin-18, Adiponectin, Reverse triiodothyronine |
| Stephen A Harrison, Mustafa | 2021 | ALT, GGT, LDL-C, HDL-C, bilirubin, ALP, glucose |
| Gi-Ae Kim | 2023 | ALT, AST, GGT, ALP, bilirubin, Triglycerides, TC, HDL-C, LDL-C, Pro-C3, CK-18, HOMA-IR, Leptin, Ghrelin, Adiponectin |

## 5. Baseline characteristics of all included literatures

Table S5：Baseline characteristics of all included literature

| Author | Publish year | Arm | Drug | Disease | Sample size | | Study period (week) | Test method | LFC Baseline (%) | Age (year) | Male (%) | White (%) | Country | Diabetes (%) | Weight (kg) | Center | Founding |
| --- | --- | --- | --- | --- | --- | --- | --- | --- | --- | --- | --- | --- | --- | --- | --- | --- | --- |
|  |  |  |  |  | Drug | Placebo |  |  |  |  |  |  |  |  |  |  |  |
| sang Bong Ahn | 2019 | 1 | multispecies probiotic mixture | NAFLD | 30 | 35 | 13 | MRI | 16.3 | 43.32 | 48.5 | / | Korea | / | 81.8 | / | Industry |
| Veeral H. Ajmera | 2019 | 1 | aramchol | NAFLD | 25 | 25 | 12 | MRI | 15.6 | 46.6 | 88 | 44 | USA | 8 | 94.7 | Multi | NA |
| Ilaria Barchetta | 2016 | 1 | vitamin.D | NAFLD | 26 | 29 | 24 | MRI | 6.8 | 57.4 | 70 | / | Italy | 100 | / | Single | Not industry |
| Eleonora Scorletti | 2020 | 1 | synbiotic treatment | NAFLD | 45 | 44 | 52 | MRS | 26.9 | 50.2 | 69 | / | UK | / | 99.2 | / | NA |
| Jeffrey Cui | 2016 | 1 | sitagliptin | NAFLD | 24 | 25 | 24 | MRI | 18.1 | 52.9 | 52 | / | USA | 48 | 92.8 | Single | Not industry |
| Samer Gawrieh | 2019 | 1 | saroglitazar | NAFLD | 26 | 28 | 16 | MRI | 20.2 | 51.1 | 50 | 84.6 | USA | / | / | Multi | Industry |
|  |  | 2 |  |  | 25 |  |  |  | 23.9 | 47.9 | 52 | 84 |  | / | / |  |  |
|  |  | 3 |  |  | 27 |  |  |  | 22.8 | 49 | 55.6 | 88.9 |  | / | / |  |  |
| Stephen A Harrison, Guy Neff | 2020 | 1 | aldafermin | NASH | 30 | 25 | 26 | MRI | 18 | 53 | 51 | 87 | USA | 62 | 102.5 | Multi | Industry |
| Stephen A Harrison | 2018 | 1 | NGM282 | NASH | 30 | 27 | 26 | MRI | 18.1 | 52 | 41 | 93 | International | 56 | 95.5 | Multi | Industry |
|  |  | 2 |  |  | 30 |  |  |  | 19.5 | 56.4 | 43 | 86 |  | 61 | 98.2 |  |  |
| Stephen A Harrison | 2021 | 1 | efruxifermin | NASH | 30 | 21 | 26 | MRI | 21.4 | 50.4 | 47 | 100 | USA | / | 108.2 | Multi | Industry |
|  |  | 2 |  |  | 30 |  |  |  | 18.3 | 52.6 | 50 | 90 |  | / | 103.6 |  |  |
|  |  | 3 |  |  | 30 |  |  |  | 19.4 | 53 | 45 | 95 |  | / | 131.1 |  |  |
| Jee‐Fu Huang‘ | 2021 | 1 | pioglitazone | NASH | 43 | 47 | 24 | MRI | 20.2 | 43.9 | 27 | 0 | China | 25.6 | / | Multi | Not industry |
| Jae Yoon Jeong | 2017 | 1 | magnolia officinalis | NAFLD | 22 | 23 | 12 | MRS | 16.12 | 39.1 | 90.9 | / | USA | / | 82.4 | Multi | NA |
|  |  | 2 |  |  | 23 |  |  |  | 12.99 | 45.5 | 60.9 | / |  | / | 78.4 | Multi |  |
| Tisha R Joy | 2016 | 1 | sitagliptin | NASH | 6 | 6 | 24 | MRI | 19 | 56.7 | 50 | 83 | USA | 100 | 100.4 |  | Not industry |
| Konstantinos Kantartzis | 2019 | 1 | resveratrol | NAFLD | 53 | 52 | 12 | MRS | 9.91 |  |  |  | USA |  |  |  | Mixed |
| W. Kim | 2017 | 1 | oltipraz | NAFLD | 21 | 21 | 21 | MRS | 30.5 | 42.6 | 71 | / | USA | / | 82.1 | Multi | Industry |
|  |  | 2 |  |  | 22 |  |  |  | 38.8 | 40.3 | 86 | / |  | / | 80.8 |  |  |
| Eunok Lee | 2019 | 1 | pinitol | NAFLD | 30 | 30 | 12 | MRI | 21.1 | 46.1 | 66.7 | / | USA | / | / | Single | Not industry |
|  |  | 2 |  |  | 30 |  |  |  | 13.7 | 45.6 | 66.7 | / |  | / | / |  |  |
| Rohit Loomba | 2020 | 1 | DGAT2 inhibitor | NAFLD | 25 | 12 | 13 | MRI | 18.5 | 64 | 52 | 100 | USA | 100 | 94.2 | Multi | Industry |
| Rohit Loomba | 2015 | 1 | ezetimibe | NASH | 23 | 21 | 24 | MRI | 15 | 49 | 44 | 76 | USA | 28 | 94.1 | Multi | Not industry |
| Atsushi Nakajima | 2021 | 1 | pemafibrate | NAFLD | 58 | 60 | 24 | MRI | 18.7 | 53.2 | 53.4 | / | USA | 31 | 80 | Multi | Industry |
| L. Pacifico | 2015 | 1 | DHA | NAFLD | 25 | 26 | 26 | MRI | 14 | 11 | 56 | / | USA | / | 66 | Single | Not industry |
| V. Ratziu | 2022 | 1 | aramchol | NASH | 90 | 48 | 52 | MRS | 27.3 | 53.9 | 36 | 62 | International | 68.3 | 88.1 | Multi | Industry |
|  |  | 2 |  |  | 83 |  |  |  | 30.2 | 54.9 | 29 | 64 | International | 67.3 | 86.9 | Multi |  |
| Vlad Ratziu | 2022 | 1 | EDP-35 | NASH | 55 | 24 | 12 | MRI | 22 | 51.5 | 47.3 | 76.4 |  | 76.4 | 96.8 | Multi | Industry |
|  | 2022 | 2 |  |  | 53 |  |  |  | 18.8 | 52.3 | 45.3 | 88.7 |  | 64.2 | 93.8 |  |  |
| Václav Šmíd | 2022 | 1 | n-3-PUFA | NAFLD | 30 | 30 | 52 | MRS | 13.44 | 51.8 | 80 | / | USA | 46.7 | 96.2 | Single | NA |
| Mark M. Smits | 2016 | 1 | liraglutide | NASH | 17 | 17 | 12 | MRS | 20.9 | 60.8 | 70.6 | / | Netherlands | 100 | 103.2 | Single | Not industry |
|  |  | 2 |  |  | 17 |  |  |  | 23.9 | 61.5 | 82.4 | / |  | 100 | 98.5 |  |  |
| Takara L Stanley | 2019 | 1 | tesamorelin | NAFLD | 31 | 26 | 52 | MRS | 12.9 | 52 | 77.4 | 67.7 | USA |  | / | Multi | Not industry |
| Norbert Stefan | 2014 | 1 | RO5093151 | NAFLD | 35 | 39 | 12 | MRS | 16.75 | 53.5 | 70 | / | International | 3 | 101.71 | Multi | Industry |
| Derek Tobin | 2018 | 1 | MF4637 | NAFLD | 81 | 86 | 24 | MRI | 14.5 | 55.3 | 44.4 | / | USA | 35 | 88.4 | Multi | Industry |
| Maneerat Chayanupatkul | 2022 | 1 | oligonol | NAFLD | 20 | 20 | 24 | MRI | 21.51 | / | / | / | Thailand | / | 78.96 | Single | Not industry |
| Sahar H. Elhini | 2022 | 1 | empagliflozin | NASH | 80 | 80 | 26 | MRI | 21.54 | / | / | / | Egypt | 100 | / | Single | Not industry |
|  |  | 2 | ursodeoxycho-lic acid | NASH | 80 |  |  |  | 19.96 | / | / | / |  | 100 | / |  |  |
| Stephen A Harrison | 2022 | 1 | aldafermin | NASH | 43 | 4 | 24 | MRI | 19.1 | 54.3 | 35 | 93 | USA | 47 | 103.4 | Multi | Industry |
|  |  | 2 |  |  | 42 |  |  |  | 17.4 | 49.8 | 31 | 90 |  | 52 | 108 |  |  |
|  |  | 3 |  |  | 43 |  |  |  | 18.7 | 52.7 | 33 | 91 |  | 56 | 109.2 |  |  |
| Thananya Jinato | 2022 | 1 | oligonol | NAFLD | 19 | 19 | 24 | MRI | 21.4 | 50.8 | 63.2 | / | Thailand | / | 78.1 | Single | Not industry |
| Rohit Loomba | 2022 | 1 | pegozafermin | NASH | 6 | 62 | 12 | MRI | 22.43 | 56.13 | 17 | 83 | USA | 83 | 87.93 | Multi | Industry |
|  |  | 2 |  |  | 12 |  |  |  | 21.43 | 49.5 | 50 | 92 |  | 33 | 87.18 |  |  |
|  |  | 3 |  |  | 11 |  |  |  | 19.3 | 51.47 | 27 | 82 |  | 64 | 87.05 |  |  |
|  |  | 4 |  |  | 10 |  |  |  | 22.01 | 51.96 | 20 | 100 |  | 40 | 94.02 |  |  |
|  |  | 5 |  |  | 14 |  |  |  | 21.57 | 51.22 | 29 | 93 |  | 21 | 101.48 |  |  |
|  |  | 6 |  |  | 9 |  |  |  | 20.94 | 52.46 | 89 | 100 |  | 22 | 101.06 |  |  |
| Aditi R. Saxena | 2023 | 1 | FP-0683591 | NAFLD | 55 | 54 | 16 | MRI | 17.5 | 58.5 | 52.7 | 78.2 | USA | 100 | 91.9 | Multi | Industry |
|  |  | 2 |  |  | 55 |  |  |  | 15.3 | 58.5 | 41.8 | 81.8 |  | 100 | 87.1 |  |  |
| Keyur | 2020 | 1 | cilofexor | NASH | 56 | 28 | 24 | MRI | 14.9 | 54 | 34 | 84 | International | 52 | 92.8 | Multi | NA |
|  |  | 2 |  |  | 56 |  |  |  | 16.1 | 58 | 38 | 75 |  | 59 | 92.4 |  |  |
| Stephen A Harrison | 2019 | 1 | resmetirom | NASH | 79 | 39 | 12 | MRI | 20.2 | 51.8 | 45 | 95 | USA | 43 | 101 | Multi | Industry |
|  |  | 2 |  |  | 79 |  |  |  | 20.2 | 51.8 | 45 | 95 |  | 43 | 101 |  |  |
| Stephen A Harrison, Mustafa | 2021 | 1 | MET409 | NASH | 19 | 19 | 12 | MRI | 18.8 | 54 | 26.3 | / | USA | 47 | 101 | Multi | Industry |
|  |  | 2 |  |  | 20 | 19 | 12 |  | 19.1 | 51 | 40 | / |  | 40 | 96 |  |  |
| Gi-Ae Kim | 2023 | 1 | ALS-L1023 | NAFLD | 19 | 20 | 24 | MRI | 16.15 | 57.1 | 63.2 | / | Korea | 47.4 | / | Multi | NA |
|  |  | 2 |  |  |  |  |  |  | 17.4 | 51.2 | 52.4 | / |  | 38.1 | / |  |  |

"Not industry" refers to trials initiated by investigators.

## 6. List of the included references

1. Ahn SB, Jun DW, Kang BK, Lim JH, Lim S, Chung MJ. Randomized, Double-blind, Placebo-controlled Study of a Multispecies Probiotic Mixture in Nonalcoholic Fatty Liver Disease. Sci Rep. 2019 Apr 5;9(1):5688. doi: 10.1038/s41598-019-42059-3. PMID: 30952918; PMCID: PMC6450966.
2. Ajmera VH, Cachay E, Ramers C, Vodkin I, Bassirian S, Singh S, Mangla N, Bettencourt R, Aldous JL, Park D, Lee D, Blanchard J, Mamidipalli A, Boehringer A, Aslam S, Leinhard OD, Richards L, Sirlin C, Loomba R. MRI Assessment of Treatment Response in HIV-associated NAFLD: A Randomized Trial of a Stearoyl-Coenzyme-A-Desaturase-1 Inhibitor (ARRIVE Trial). Hepatology. 2019 Nov;70(5):1531-1545. doi: 10.1002/hep.30674. Epub 2019 Jun 18. PMID: 31013363; PMCID: PMC7164416.
3. Barchetta I, Del Ben M, Angelico F, Di Martino M, Fraioli A, La Torre G, Saulle R, Perri L, Morini S, Tiberti C, Bertoccini L, Cimini FA, Panimolle F, Catalano C, Baroni MG, Cavallo MG. No effects of oral vitamin D supplementation on non-alcoholic fatty liver disease in patients with type 2 diabetes: a randomized, double-blind, placebo-controlled trial. BMC Med. 2016 Jun 29;14:92. doi: 10.1186/s12916-016-0638-y. PMID: 27353492; PMCID: PMC4926287.
4. Scorletti E, Afolabi PR, Miles EA, Smith DE, Almehmadi A, Alshathry A, Childs CE, Del Fabbro S, Bilson J, Moyses HE, Clough GF, Sethi JK, Patel J, Wright M, Breen DJ, Peebles C, Darekar A, Aspinall R, Fowell AJ, Dowman JK, Nobili V, Targher G, Delzenne NM, Bindels LB, Calder PC, Byrne CD. Synbiotics Alter Fecal Microbiomes, But Not Liver Fat or Fibrosis, in a Randomized Trial of Patients With Nonalcoholic Fatty Liver Disease. Gastroenterology. 2020 May;158(6):1597-1610.e7. doi: 10.1053/j.gastro.2020.01.031. Epub 2020 Jan 25. PMID: 31987796; PMCID: PMC7613160.
5. Cui J, Philo L, Nguyen P, Hofflich H, Hernandez C, Bettencourt R, Richards L, Salotti J, Bhatt A, Hooker J, Haufe W, Hooker C, Brenner DA, Sirlin CB, Loomba R. Sitagliptin vs. placebo for non-alcoholic fatty liver disease: A randomized controlled trial. J Hepatol. 2016 Aug;65(2):369-76. doi: 10.1016/j.jhep.2016.04.021. Epub 2016 May 2. PMID: 27151177; PMCID: PMC5081213.
6. Gawrieh S, Noureddin M, Loo N, Mohseni R, Awasty V, Cusi K, Kowdley KV, Lai M, Schiff E, Parmar D, Patel P, Chalasani N. Saroglitazar, a PPAR-α/γ Agonist, for Treatment of NAFLD: A Randomized Controlled Double-Blind Phase 2 Trial. Hepatology. 2021 Oct;74(4):1809-1824. doi: 10.1002/hep.31843. Epub 2021 Jul 19. PMID: 33811367.
7. Harrison SA, Neff G, Guy CD, Bashir MR, Paredes AH, Frias JP, Younes Z, Trotter JF, Gunn NT, Moussa SE, Kohli A, Nelson K, Gottwald M, Chang WCG, Yan AZ, DePaoli AM, Ling L, Lieu HD. Efficacy and Safety of Aldafermin, an Engineered FGF19 Analog, in a Randomized, Double-Blind, Placebo-Controlled Trial of Patients With Nonalcoholic Steatohepatitis. Gastroenterology. 2021 Jan;160(1):219-231.e1. doi: 10.1053/j.gastro.2020.08.004. Epub 2020 Aug 8. PMID: 32781086.
8. Harrison SA, Rinella ME, Abdelmalek MF, Trotter JF, Paredes AH, Arnold HL, Kugelmas M, Bashir MR, Jaros MJ, Ling L, Rossi SJ, DePaoli AM, Loomba R. NGM282 for treatment of non-alcoholic steatohepatitis: a multicentre, randomised, double-blind, placebo-controlled, phase 2 trial. Lancet. 2018 Mar 24;391(10126):1174-1185. doi: 10.1016/S0140-6736(18)30474-4. Epub 2018 Mar 5. Erratum in: Lancet. 2018 Mar 24;391(10126):e16. PMID: 29519502.
9. Harrison SA, Ruane PJ, Freilich BL, Neff G, Patil R, Behling CA, Hu C, Fong E, de Temple B, Tillman EJ, Rolph TP, Cheng A, Yale K. Efruxifermin in non-alcoholic steatohepatitis: a randomized, double-blind, placebo-controlled, phase 2a trial. Nat Med. 2021 Jul;27(7):1262-1271. doi: 10.1038/s41591-021-01425-3. Epub 2021 Jul 8. PMID: 34239138.
10. Huang JF, Dai CY, Huang CF, Tsai PC, Yeh ML, Hsu PY, Huang SF, Bair MJ, Hou NJ, Huang CI, Liang PC, Lin YH, Wang CW, Hsieh MY, Chen SC, Lin ZY, Yu ML, Chuang WL. First-in-Asian double-blind randomized trial to assess the efficacy and safety of insulin sensitizer in nonalcoholic steatohepatitis patients. Hepatol Int. 2021 Oct;15(5):1136-1147. doi: 10.1007/s12072-021-10242-2. Epub 2021 Aug 12. PMID: 34386935.
11. Jeong JY, Sohn JH, Baek YH, Cho YK, Kim Y, Kim H. New botanical drug, HL tablet, reduces hepatic fat as measured by magnetic resonance spectroscopy in patients with nonalcoholic fatty liver disease: A placebo-controlled, randomized, phase II trial. World J Gastroenterol. 2017 Aug 28;23(32):5977-5985. doi: 10.3748/wjg.v23.i32.5977. PMID: 28932090; PMCID: PMC5583583.
12. Joy TR, McKenzie CA, Tirona RG, Summers K, Seney S, Chakrabarti S, Malhotra N, Beaton MD. Sitagliptin in patients with non-alcoholic steatohepatitis: A randomized, placebo-controlled trial. World J Gastroenterol. 2017 Jan 7;23(1):141-150. doi: 10.3748/wjg.v23.i1.141. PMID: 28104990; PMCID: PMC5221278.
13. Kantartzis K, Fritsche L, Bombrich M, Machann J, Schick F, Staiger H, Kunz I, Schoop R, Lehn-Stefan A, Heni M, Peter A, Fritsche A, Häring HU, Stefan N. Effects of resveratrol supplementation on liver fat content in overweight and insulin-resistant subjects: A randomized, double-blind, placebo-controlled clinical trial. Diabetes Obes Metab. 2018 Jul;20(7):1793-1797. doi: 10.1111/dom.13268. Epub 2018 Mar 22. PMID: 29484808.
14. Kim W, Kim BG, Lee JS, Lee CK, Yeon JE, Chang MS, Kim JH, Kim H, Yi S, Lee J, Cho JY, Kim SG, Lee JH, Kim YJ. Randomised clinical trial: the efficacy and safety of oltipraz, a liver X receptor alpha-inhibitory dithiolethione in patients with non-alcoholic fatty liver disease. Aliment Pharmacol Ther. 2017 Apr;45(8):1073-1083. doi: 10.1111/apt.13981. Epub 2017 Feb 22. PMID: 28225186.
15. Lee E, Lim Y, Kwon SW, Kwon O. Pinitol consumption improves liver health status by reducing oxidative stress and fatty acid accumulation in subjects with non-alcoholic fatty liver disease: A randomized, double-blind, placebo-controlled trial. J Nutr Biochem. 2019 Jun;68:33-41. doi: 10.1016/j.jnutbio.2019.03.006. Epub 2019 Mar 28. PMID: 31030165.
16. Loomba R, Morgan E, Watts L, Xia S, Hannan LA, Geary RS, Baker BF, Bhanot S. Novel antisense inhibition of diacylglycerol O-acyltransferase 2 for treatment of non-alcoholic fatty liver disease: a multicentre, double-blind, randomised, placebo-controlled phase 2 trial. Lancet Gastroenterol Hepatol. 2020 Sep;5(9):829-838. doi: 10.1016/S2468-1253(20)30186-2. Epub 2020 Jun 15. PMID: 32553151.
17. Loomba R, Sirlin CB, Ang B, Bettencourt R, Jain R, Salotti J, Soaft L, Hooker J, Kono Y, Bhatt A, Hernandez L, Nguyen P, Noureddin M, Haufe W, Hooker C, Yin M, Ehman R, Lin GY, Valasek MA, Brenner DA, Richards L; San Diego Integrated NAFLD Research Consortium (SINC). Ezetimibe for the treatment of nonalcoholic steatohepatitis: assessment by novel magnetic resonance imaging and magnetic resonance elastography in a randomized trial (MOZART trial). Hepatology. 2015 Apr;61(4):1239-50. doi: 10.1002/hep.27647. Epub 2015 Feb 27. PMID: 25482832; PMCID: PMC4407930.
18. Nakajima A, Eguchi Y, Yoneda M, Imajo K, Tamaki N, Suganami H, Nojima T, Tanigawa R, Iizuka M, Iida Y, Loomba R. Randomised clinical trial: Pemafibrate, a novel selective peroxisome proliferator-activated receptor α modulator (SPPARMα), versus placebo in patients with non-alcoholic fatty liver disease. Aliment Pharmacol Ther. 2021 Nov;54(10):1263-1277. doi: 10.1111/apt.16596. Epub 2021 Sep 16. PMID: 34528723; PMCID: PMC9292296.
19. Pacifico L, Bonci E, Di Martino M, Versacci P, Andreoli G, Silvestri LM, Chiesa C. A double-blind, placebo-controlled randomized trial to evaluate the efficacy of docosahexaenoic acid supplementation on hepatic fat and associated cardiovascular risk factors in overweight children with nonalcoholic fatty liver disease. Nutr Metab Cardiovasc Dis. 2015 Aug;25(8):734-41. doi: 10.1016/j.numecd.2015.04.003. Epub 2015 Apr 25. PMID: 26026214.
20. Ratziu V, de Guevara L, Safadi R, Poordad F, Fuster F, Flores-Figueroa J, Arrese M, Fracanzani AL, Ben Bashat D, Lackner K, Gorfine T, Kadosh S, Oren R, Halperin M, Hayardeny L, Loomba R, Friedman S; ARREST investigator study group; Sanyal AJ. Aramchol in patients with nonalcoholic steatohepatitis: a randomized, double-blind, placebo-controlled phase 2b trial. Nat Med. 2021 Oct;27(10):1825-1835. doi: 10.1038/s41591-021-01495-3. Epub 2021 Oct 7. PMID: 34621052.
21. Ratziu V, Rinella ME, Neuschwander-Tetri BA, Lawitz E, Denham D, Kayali Z, Sheikh A, Kowdley KV, Desta T, Elkhashab M, DeGrauw J, Goodwin B, Ahmad A, Adda N. EDP-305 in patients with NASH: A phase II double-blind placebo-controlled dose-ranging study. J Hepatol. 2022 Mar;76(3):506-517. doi: 10.1016/j.jhep.2021.10.018. Epub 2021 Nov 3. PMID: 34740705.
22. Šmíd V, Dvořák K, Šedivý P, Kosek V, Leníček M, Dezortová M, Hajšlová J, Hájek M, Vítek L, Bechyňská K, Brůha R. Effect of Omega-3 Polyunsaturated Fatty Acids on Lipid Metabolism in Patients With Metabolic Syndrome and NAFLD. Hepatol Commun. 2022 Jun;6(6):1336-1349. doi: 10.1002/hep4.1906. Epub 2022 Feb 11. PMID: 35147302; PMCID: PMC9134818.
23. Smits MM, Tonneijck L, Muskiet MH, Kramer MH, Pouwels PJ, Pieters-van den Bos IC, Hoekstra T, Diamant M, van Raalte DH, Cahen DL. Twelve week liraglutide or sitagliptin does not affect hepatic fat in type 2 diabetes: a randomised placebo-controlled trial. Diabetologia. 2016 Dec;59(12):2588-2593. doi: 10.1007/s00125-016-4100-7. Epub 2016 Sep 15. PMID: 27627981; PMCID: PMC6518065.
24. Stanley TL, Fourman LT, Feldpausch MN, Purdy J, Zheng I, Pan CS, Aepfelbacher J, Buckless C, Tsao A, Kellogg A, Branch K, Lee H, Liu CY, Corey KE, Chung RT, Torriani M, Kleiner DE, Hadigan CM, Grinspoon SK. Effects of tesamorelin on non-alcoholic fatty liver disease in HIV: a randomised, double-blind, multicentre trial. Lancet HIV. 2019 Dec;6(12):e821-e830. doi: 10.1016/S2352-3018(19)30338-8. Epub 2019 Oct 11. PMID: 31611038; PMCID: PMC6981288.
25. Stefan N, Ramsauer M, Jordan P, Nowotny B, Kantartzis K, Machann J, Hwang JH, Nowotny P, Kahl S, Harreiter J, Hornemann S, Sanyal AJ, Stewart PM, Pfeiffer AF, Kautzky-Willer A, Roden M, Häring HU, Fürst-Recktenwald S. Inhibition of 11β-HSD1 with RO5093151 for non-alcoholic fatty liver disease: a multicentre, randomised, double-blind, placebo-controlled trial. Lancet Diabetes Endocrinol. 2014 May;2(5):406-16. doi: 10.1016/S2213-8587(13)70170-0. Epub 2014 Feb 17. PMID: 24795254.
26. Tobin D, Brevik-Andersen M, Qin Y, Innes JK, Calder PC. Evaluation of a High Concentrate Omega-3 for Correcting the Omega-3 Fatty Acid Nutritional Deficiency in Non-Alcoholic Fatty Liver Disease (CONDIN). Nutrients. 2018 Aug 20;10(8):1126. doi: 10.3390/nu10081126. PMID: 30127297; PMCID: PMC6115838.
27. Chayanupatkul M, Sawatdee W, Chutaputti A, Tangkijvanich P. The Efficacy of Oligonol in Nonalcoholic Fatty Liver Disease: A Randomized Double-Blinded Placebo-Controlled Trial. J Integr Complement Med. 2022 Nov;28(11):904-908. doi: 10.1089/jicm.2021.0362. Epub 2022 Sep 7. PMID: 36074799.
28. Elhini SH, Wahsh EA, Elberry AA, El Ameen NF, Abdelfadil Saedii A, Refaie SM, Elsayed AA, Rabea HM. The Impact of an SGLT2 Inhibitor versus Ursodeoxycholic Acid on Liver Steatosis in Diabetic Patients. Pharmaceuticals (Basel). 2022 Dec 5;15(12):1516. doi: 10.3390/ph15121516. PMID: 36558967; PMCID: PMC9786599.
29. Harrison SA, Abdelmalek MF, Neff G, Gunn N, Guy CD, Alkhouri N, Bashir MR, Freilich B, Kohli A, Khazanchi A, Sheikh MY, Leibowitz M, Rinella ME, Siddiqui MS, Kipnes M, Moussa SE, Younes ZH, Bansal M, Baum SJ, Borg B, Ruane PJ, Thuluvath PJ, Gottwald M, Khan M, Chen C, Melchor-Khan L, Chang W, DePaoli AM, Ling L, Lieu HD. Aldafermin in patients with non-alcoholic steatohepatitis (ALPINE 2/3): a randomised, double-blind, placebo-controlled, phase 2b trial. Lancet Gastroenterol Hepatol. 2022 Jul;7(7):603-616. doi: 10.1016/S2468-1253(22)00017-6. Epub 2022 Mar 21. PMID: 35325622.
30. Jinato T, Chayanupatkul M, Dissayabutra T, Chutaputti A, Tangkijvanich P, Chuaypen N. Litchi-Derived Polyphenol Alleviates Liver Steatosis and Gut Dysbiosis in Patients with Non-Alcoholic Fatty Liver Disease: A Randomized Double-Blinded, Placebo-Controlled Study. Nutrients. 2022 Jul 16;14(14):2921. doi: 10.3390/nu14142921. PMID: 35889878; PMCID: PMC9319370.
31. Loomba R, Lawitz EJ, Frias JP, Ortiz-Lasanta G, Johansson L, Franey BB, Morrow L, Rosenstock M, Hartsfield CL, Chen CY, Tseng L, Charlton RW, Mansbach H, Margalit M. Safety, pharmacokinetics, and pharmacodynamics of pegozafermin in patients with non-alcoholic steatohepatitis: a randomised, double-blind, placebo-controlled, phase 1b/2a multiple-ascending-dose study. Lancet Gastroenterol Hepatol. 2023 Feb;8(2):120-132. doi: 10.1016/S2468-1253(22)00347-8. Epub 2022 Dec 12. PMID: 36521501.
32. Saxena AR, Lyle SA, Khavandi K, Qiu R, Whitlock M, Esler WP, Kim AM. A phase 2a, randomized, double-blind, placebo-controlled, three-arm, parallel-group study to assess the efficacy, safety, tolerability and pharmacodynamics of PF-06835919 in patients with non-alcoholic fatty liver disease and type 2 diabetes. Diabetes Obes Metab. 2023 Apr;25(4):992-1001. doi: 10.1111/dom.14946. Epub 2023 Jan 17. PMID: 36515213.
33. Patel K, Harrison SA, Elkhashab M, Trotter JF, Herring R, Rojter SE, Kayali Z, Wong VW, Greenbloom S, Jayakumar S, Shiffman ML, Freilich B, Lawitz EJ, Gane EJ, Harting E, Xu J, Billin AN, Chung C, Djedjos CS, Subramanian GM, Myers RP, Middleton MS, Rinella M, Noureddin M. Cilofexor, a Nonsteroidal FXR Agonist, in Patients With Noncirrhotic NASH: A Phase 2 Randomized Controlled Trial. Hepatology. 2020 Jul;72(1):58-71. doi: 10.1002/hep.31205. PMID: 32115759.
34. Harrison SA, Bashir MR, Guy CD, Zhou R, Moylan CA, Frias JP, Alkhouri N, Bansal MB, Baum S, Neuschwander-Tetri BA, Taub R, Moussa SE. Resmetirom (MGL-3196) for the treatment of non-alcoholic steatohepatitis: a multicentre, randomised, double-blind, placebo-controlled, phase 2 trial. Lancet. 2019 Nov 30;394(10213):2012-2024. doi: 10.1016/S0140-6736(19)32517-6. Epub 2019 Nov 11. PMID: 31727409.
35. Harrison SA, Bashir MR, Lee KJ, Shim-Lopez J, Lee J, Wagner B, Smith ND, Chen HC, Lawitz EJ. A structurally optimized FXR agonist, MET409, reduced liver fat content over 12 weeks in patients with non-alcoholic steatohepatitis. J Hepatol. 2021 Jul;75(1):25-33. doi: 10.1016/j.jhep.2021.01.047. Epub 2021 Feb 11. PMID: 33581174.
36. Kim GA, Cho HC, Jeong SW, Kang BK, Kim M, Jung S, Hwang J, Yoon EL, Jun DW. A Phase 2a, Randomized, Double-Blind, Placebo-Controlled Study to Assess the Efficacy and Safety of ALS-L1023 in Non-Alcoholic Fatty Liver Disease. Pharmaceuticals (Basel). 2023 Apr 20;16(4):623. doi: 10.3390/ph16040623. PMID: 37111380; PMCID: PMC10142612.

## 7. Details of modeling analysis

**Establishment of the base model**

Exploratory data analysis revealed that the relationships between ΔΔAST or ΔΔALT and ΔΔLFC exhibit linear characteristics. Therefore, a linear model was employed to describe the relationships between ΔΔLFC and ΔΔALT or ΔΔAST.

ΔΔLFC=α_1_×ΔΔALT-β_1_ Equation 7.1

ΔΔLFC=α_2_×ΔΔAST-β_2_ Equation 7.2

In Equations 8.1 and 8.2, α_1_ and α_2_ represent the slope parameters, while β_1_ and β_2_ are the intercept parameters.

### Covariate analysis

This analysis explores factors that could influence the model parameters by establishing a covariate model that includes variables such as age and baseline characteristics. For covariates with a missing data rate less than 30%, missing values are imputed using the median. Covariates with a missing rate exceeding 30% are excluded from the model. Binary covariates are incorporated using Equation 7.3, while continuous covariates are introduced using Equations 8.4 and 8.5.

$P_{i}=P_{Typical}+COV\times\theta_{cov}$ Equation 7.3

$P_{i}=P_{Typical}+(COV-{COV}_{median})\times\theta_{cov}$ Equation 7.4

$P_{i}=P_{Typical}\times{(\frac{COV}{{COV}_{median}})}^{\theta_{cov}}$ Equation 7.5

In Formulas 7.1-7.3, *P_i_* represents the model parameters at different levels of covariates, *P_Typical_* represents the typical values of the model parameters. COV represents the covariate value, *COV_median_* represents the median of the continuous covariate, and *θ_cov_* represents the correction coefficient of the introduced covariate on the model parameters.

The covariates that significantly impact the model are selected using a stepwise covariate method, the threshold for forward inclusion is set at 3.84, corresponding to a significance level of *P< .05*, while the threshold for backward exclusion is established at 6.63, indicative of a significance level of *P< .01*.

### Model evaluation

The model's initial evaluation involves analyzing the standard errors of the model parameters and the objective function value (OFV). The bootstrap method is used to assess the stability of the model, by comparing the distribution of model parameters derived from 1000 repeated samples to those of the original parameters. The VPC evaluates the predictive performance of the model by simulating 1000 datasets and comparing the observed efficacy values against the 2.5^th^ percentile, median, and 97.5^th^ percentile of the predicted value.

## 8. Missing SD data imputation method

For the imputation of missing standard deviation (SD) data, please refer to the official Cochrane website guide ([https://training.cochrane.org/handbook/current/](https://training.cochrane.org/handbook/current%20/) chapter-23). The calculation formula can be found in Equation 9.1.


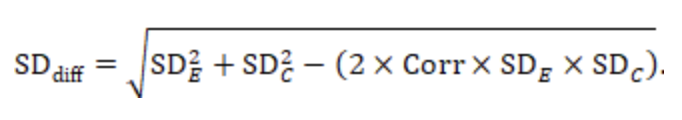
 Equation 8.1

In Equation 8.1, SD_diff_ represents the standard deviation of the difference between the drug and control groups. SD_E_ and SD_c_ correspond to the standard deviations observed in the drug and control groups, respectively. Corr denotes the correlation coefficient, which can be estimated from literature that reports these standard deviation parameters. In instances where the literature does not report specific standard deviation values, imputation is performed using median values from other relevant studies.

In the reviewed literature, out of the total, 12 articles (33%) reported the standard deviation (SD) of changes in liver fat content (LFC) relative to baseline. Another 13 articles (36%) provided the SDs for both baseline and endpoint, from which the SDs for the changes were calculated. The SDs for the remaining 11 articles (31%) were imputed based on the median.

## 9. Risk of bias

Among the studies evaluated, 16 were identified as having a 'high' risk of bias, 5 with a 'medium' risk, and 15 with a 'low' risk, as illustrated in Figure S9. Factors contributing to a high risk of bias included unclear management of missing data in intention-to-treat (ITT) analyses, the absence of measures such as LFC in registered protocols suggesting potential selective reporting, and the execution of post-hoc analyses. Medium risk was linked to unlocated registered protocols and significant baseline differences between the treatment and placebo groups.


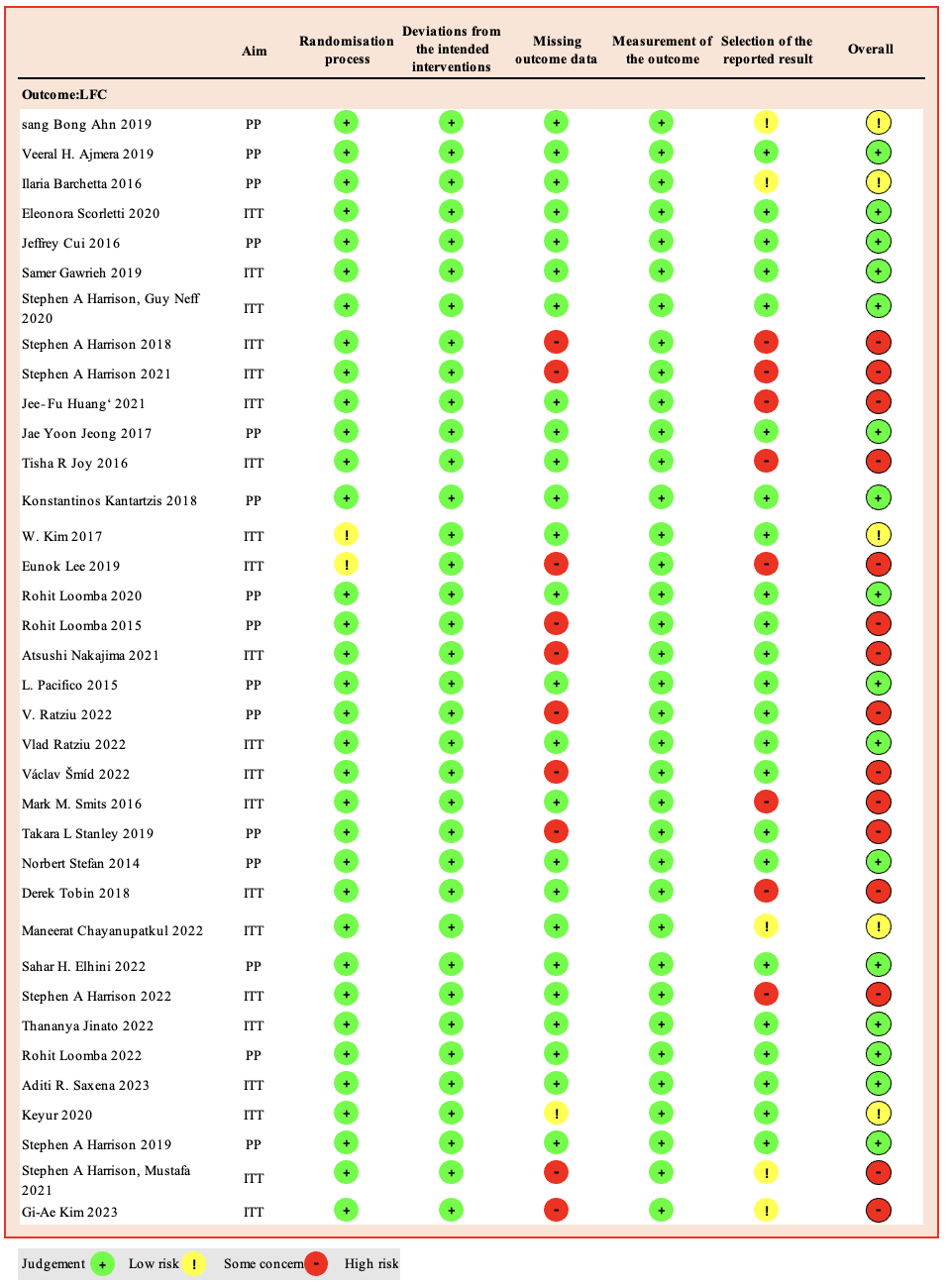


Figure S9. Risk of bias of the included literature

10. Meta-analysis results for ALT, AST, and LFC


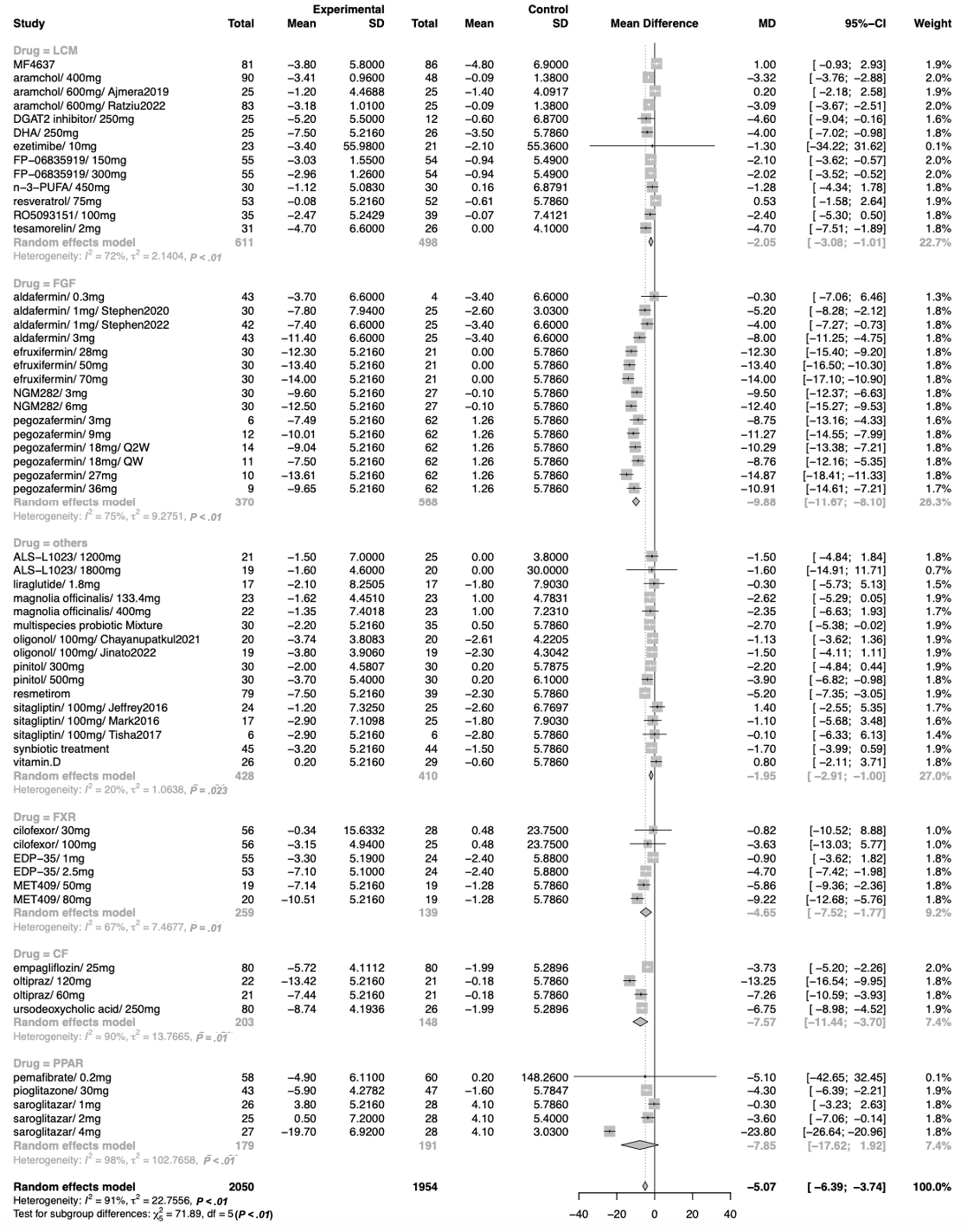


Figure S10.1：Forest plot of △△LFC

LCM: Drugs acting on glucose and lipid metabolism, FGF: FGF agonist, FXR: FXR agonist, CF: Drugs acting on cell death, inflammation, and fibrosis, PPAR: PPAR agonist.


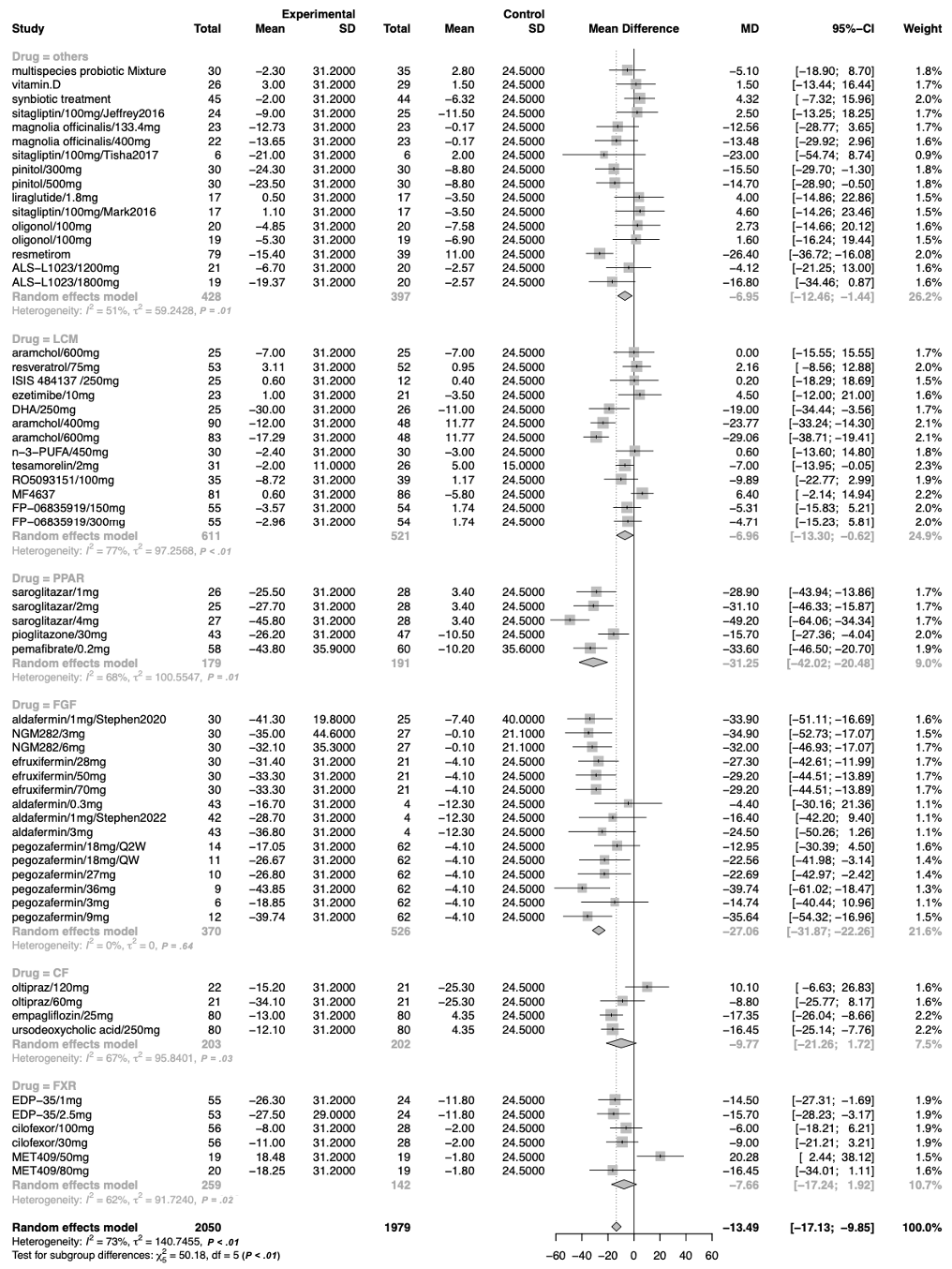


Figure S10.2：Forest plot of △△ALT


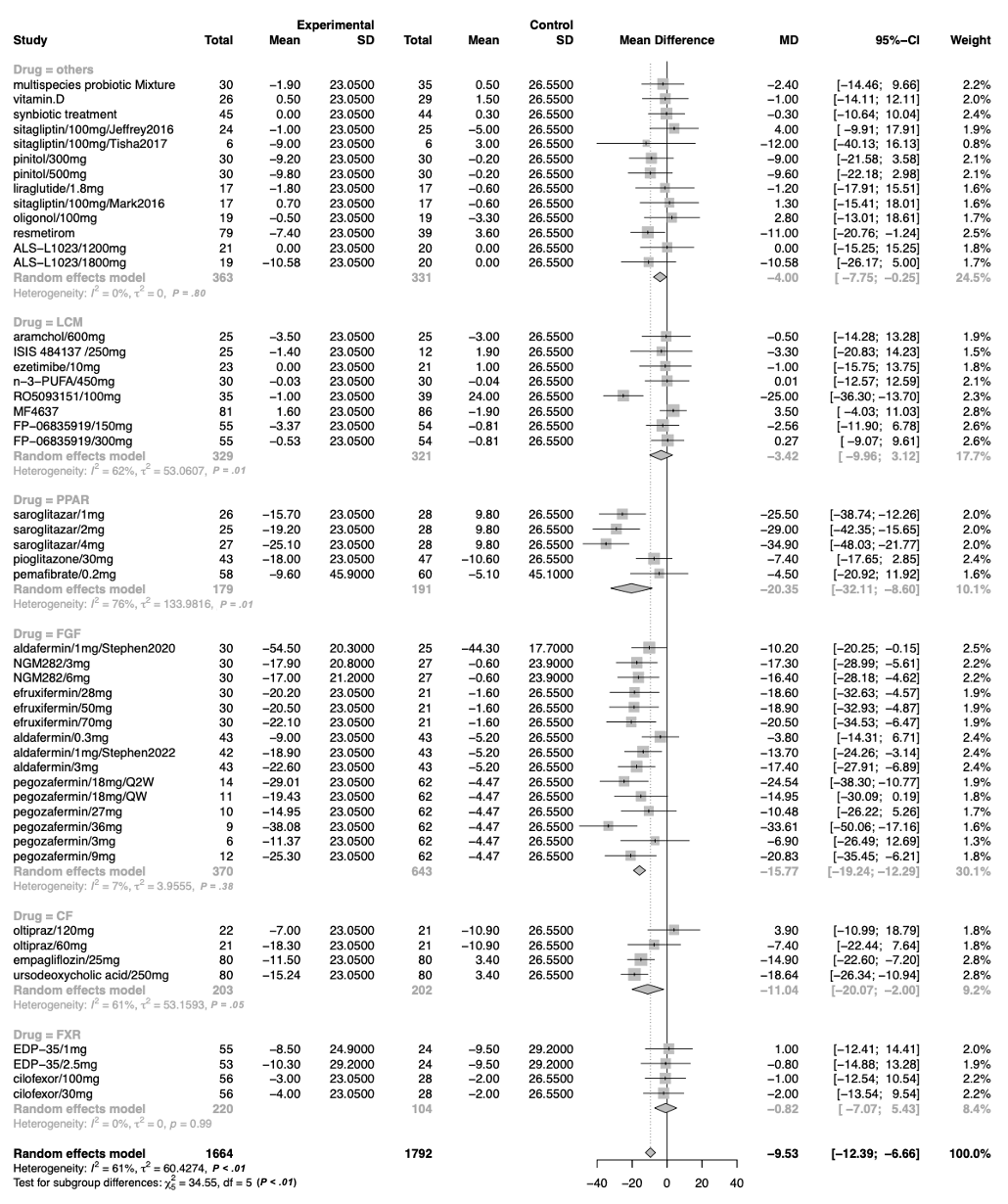


Figure S10.3：Forest plot of △△AST

## 11. Results of Subgroup analysis

Table S11 Comparison of subgroup analysis results of △△LFC with original findings

| **Drugs** | **Original data** | **Excluding highly biased literature** | **Excluding MRS-PDFF detected data** | **Excluding therapies with only a single literature** |
| --- | --- | --- | --- | --- |
| LCM | -2.05 (-3.08, -1.01), I^2^=72% | -2.08 (-3.22, -0.94), I^2^=71% | -1.58 (-3.12, -0.03), I^2^=57% | -2.48 (-3.4, -1.56), I^2^=67% |
| FGF | -9.88 (-11.67, -8.1), I^2^=75% | -9.22 (-11.8, -6.64), I^2^=73% | -9.88 (-11.67, -8.1), I^2^=75% | -9.88 (-11.67, -8.1), I^2^=75% |
| FXR | -4.65 (-7.52, -1.77), I^2^=67% | -2.72 (-5.54, 0.11), I^2^=24% | -4.65 (-7.52, -1.77), I^2^=67% | -4.65 (-7.52, -1.77), I^2^=67% |
| CF | -7.57 (-11.44, -3.7), I^2^=90% | -7.57 (-11.44, -3.7), I^2^=90% | -5.12 (-8.07, -2.17), I^2^=80% | -10.26 (-16.12, -4.4), I^2^=84% |
| PPAR | -7.85 (-17.62, 1.92), I^2^=98% | -9.25 (-23.7, 5.17), I^2^=99% | -7.85 (-17.62, 1.92), I^2^=98% | -7.85 (-17.62, 1.92), I^2^=98% |
| others | -1.95 (-2.91, -1), I^2^=20% | -1.87 (-3.17, -0.57), I^2^=50% | -1.96 (-3.18, -0.74), I^2^=38% | -1.79 (-2.79, -0.79), I^2^=0% |
| All drugs | -5.07 (-6.39, -3.74), I^2^=91% | -4.81 (-6.55, -3.08), I^2^=92% | -5.53 (-7.1, -3.96), I^2^=91% | -5.94 (-7.58, -4.3), I^2^=92% |


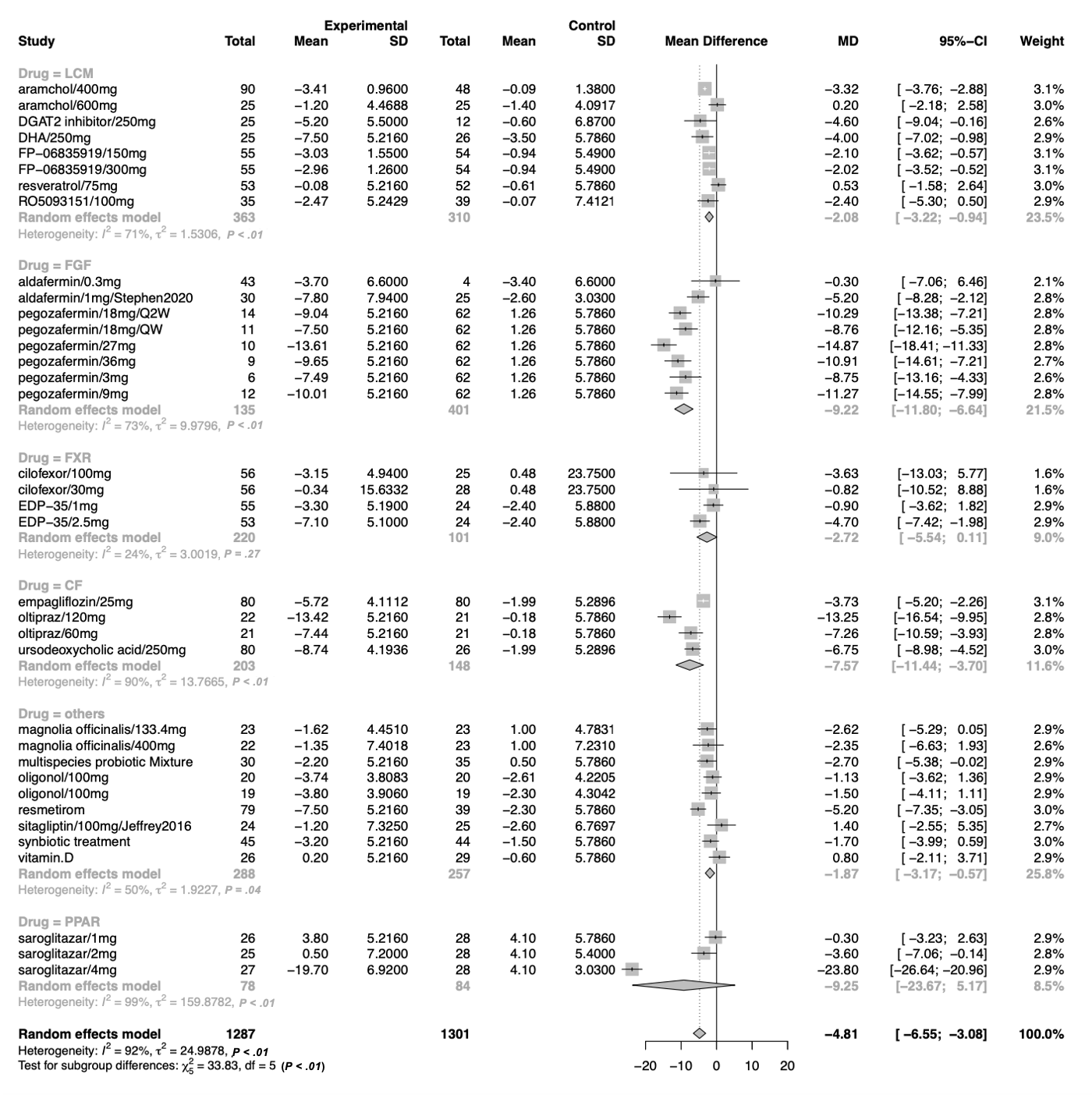


Figure S11.1: forest plot of △△LFC after excluding highly biased literature

LCM: Drugs acting on glucose and lipid metabolism, FGF: FGF agonist, FXR: FXR agonist, CF: Drugs acting on cell death, inflammation, and fibrosis, PPAR: PPAR agonist.


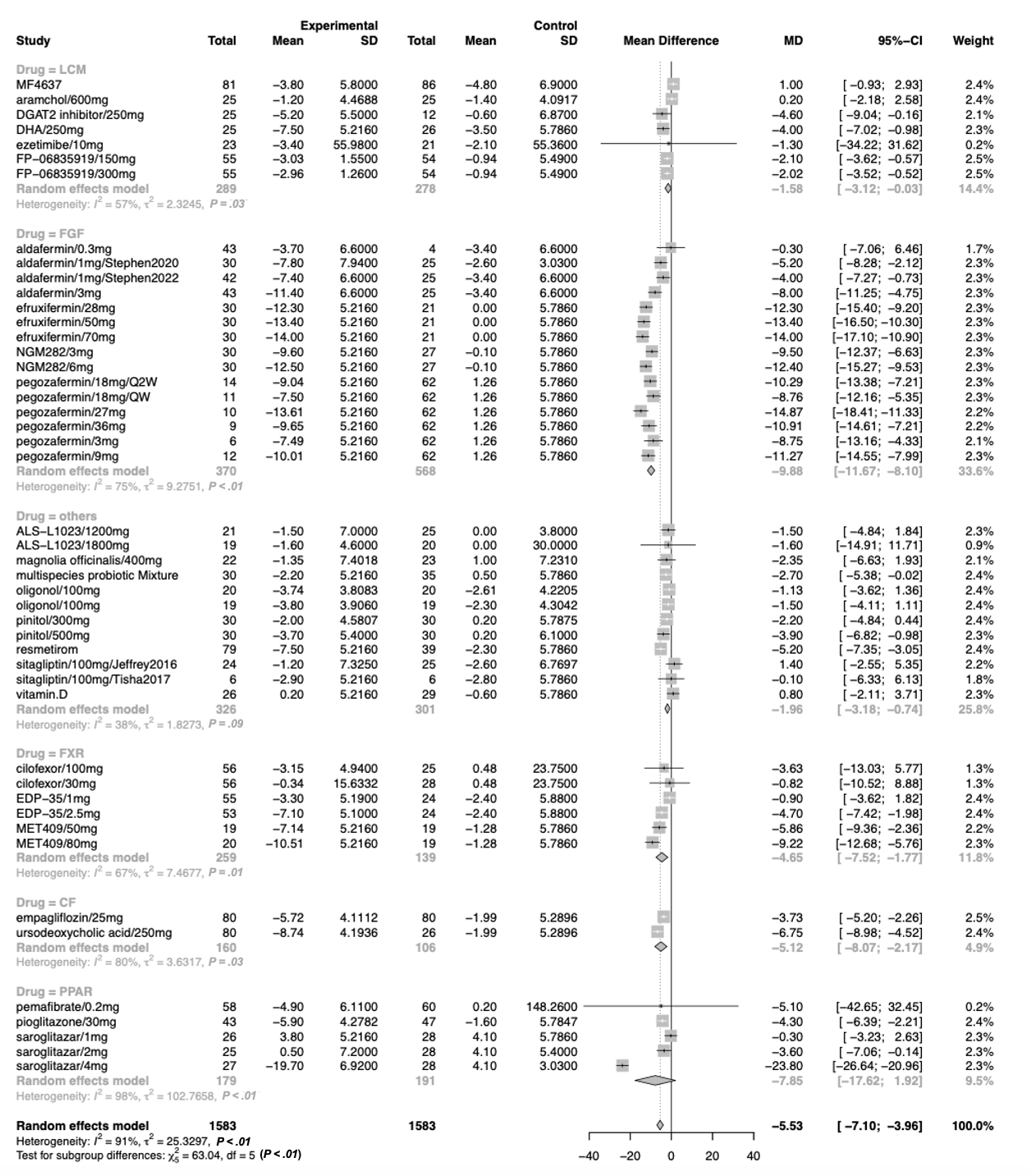


Figure S11.2 Forest plot of △△LFC after excluding MRS-PDFF detected data


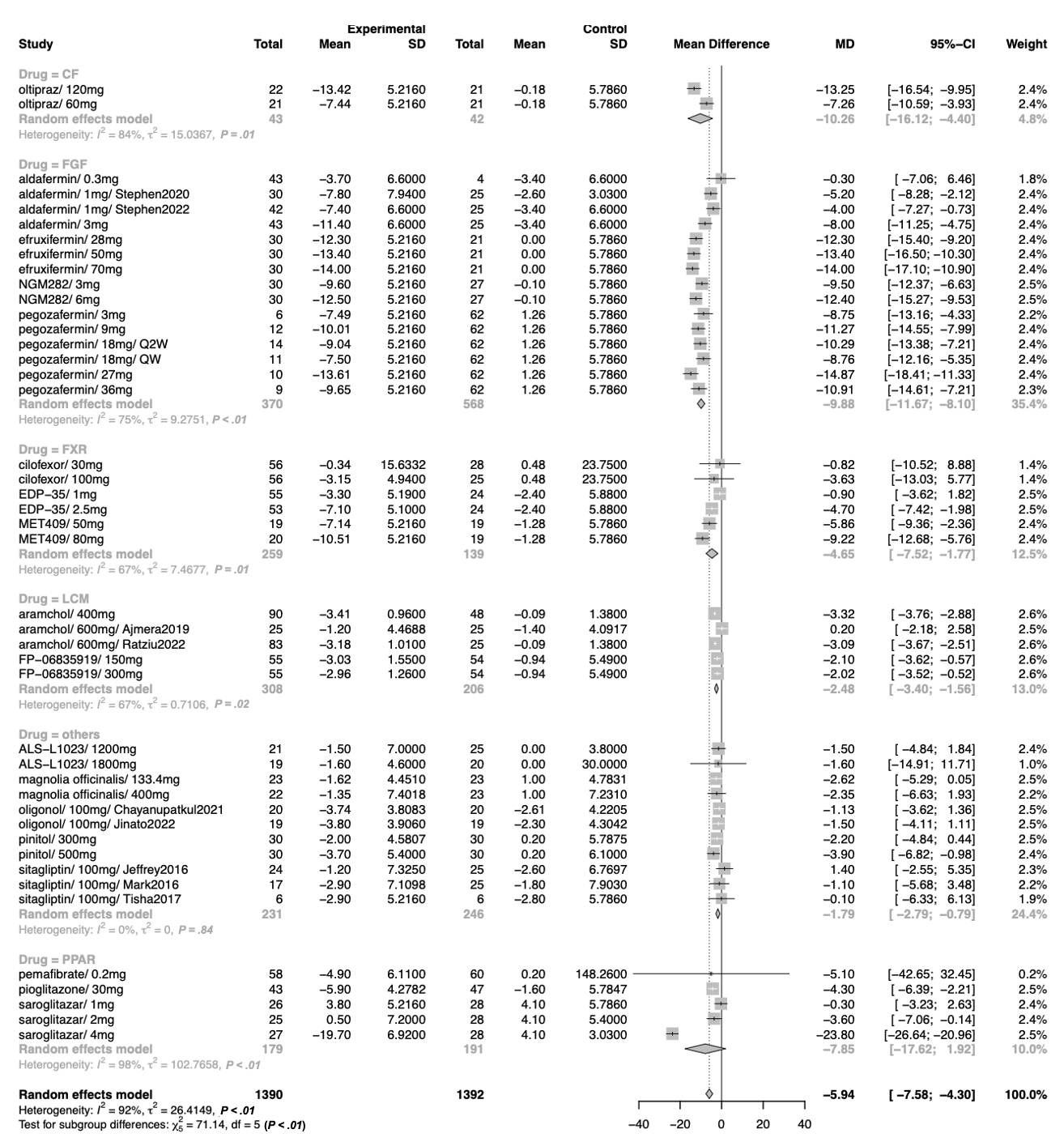


Figure S11.3 Forest plot of △△LFC after excluding the therapies with only a single literature

## 12. Time-course characteristics of △△LFC


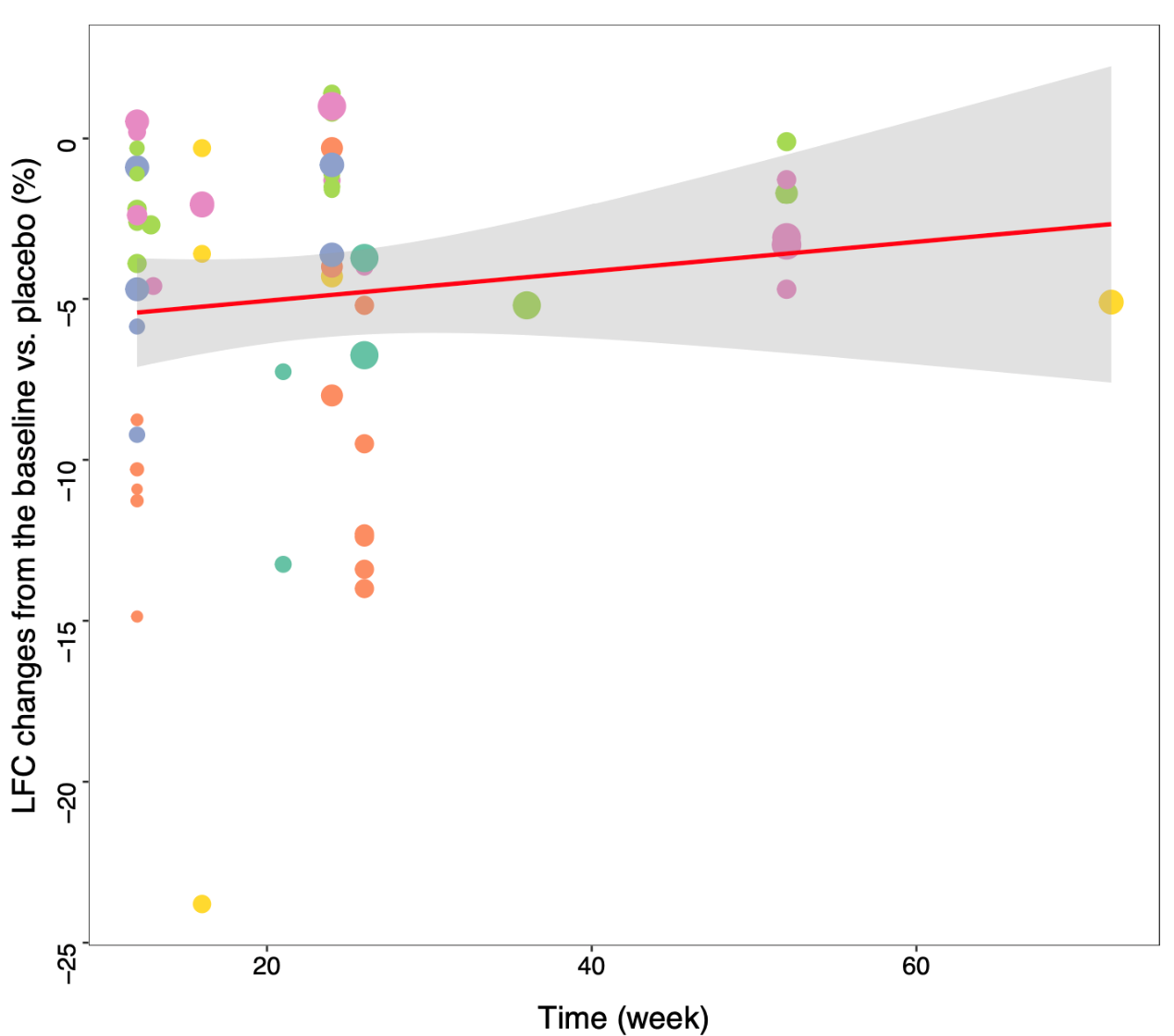


Figure S12: Distribution of ΔΔLFC at different time points

## 13. Baseline characteristic of the included literature

Table S13: Baseline characteristics of the included literature

| **Characteristic** | **Median (min, max)**  **arm=59** | **Reporting rate (%)** |
| --- | --- | --- |
| Sample size | 30 (6, 90) | 100 |
| Trial period | 21 (12, 52) | 100 |
| Detecte methods |  |  |
| MRI |  | 78 |
| MRS |  | 22 |
| Age, years | 52 (11, 64) | 93.2 |
| Male, % | 50 (17, 90.9) | 93.2 |
| Race |  |  |
| White, % | 86.5 (0, 100) | 55.9 |
| Region |  |  |
| International studies |  | 15.3 |
| Asia |  | 11.9 |
| America |  | 59.3 |
| Europe |  | 13.6 |
| Weight (kg) | 94.2 (66, 131.1) | 79.7 |
| Body mass index (kg/m^2^) | 32.9 (24.3, 38.8) | 93.2 |
| Liver fat content % | 19.1 (6.8, 38.8) | 100 |
| Liver chemistry |  |  |
| ALT, U/L | 56.3 (23.4, 105.4) | 100 |
| AST, U/L | 39.0 (11.5, 58.4) | 89.8 |
| GGT, U/L | 60.6 (34, 164) | 69.5 |
| Lipid panel |  |  |
| Triglycerides, mg/dl | 151 (92, 210.8) | 55.9 |
| Cholesterol, mg/dl | 191 (152, 224.6) | 52.5 |
| Metabolic parameters |  |  |
| HOMA-IR | 6.6 (2.3, 93.2) | 19.4 |
| Glucose, mmol/l | 116.7 (83, 149.5) | 39 |
| Insulin, μIU/ml | 20.9 (11.7, 37.4) | 33.9 |
| HbA1c, % | 6.8 (5.7, 9.0) | 33.9 |
| Multicenter |  | 71.2 |
| Single center |  | 28.8 |
| The time of publication | 2020 (2014, 2023) | 100 |

## 14. List of excluded literatures after full-text reading

| **ID** | **Reference** |
| --- | --- |
| 1 | Bhatia L, Scorletti E, Curzen N, Clough GF, Calder PC, Byrne CD. Improvement in non-alcoholic fatty liver disease severity is associated with a reduction in carotid intima-media thickness progression. Atherosclerosis. 2016 Mar;246:13-20. doi: 10.1016/j.atherosclerosis.2015.12.028. Epub 2015 Dec 24. PMID: 26748347. |
| 2 | Chachay VS, Macdonald GA, Martin JH, Whitehead JP, O'Moore-Sullivan TM, Lee P, Franklin M, Klein K, Taylor PJ, Ferguson M, Coombes JS, Thomas GP, Cowin GJ, Kirkpatrick CM, Prins JB, Hickman IJ. Resveratrol does not benefit patients with nonalcoholic fatty liver disease. Clin Gastroenterol Hepatol. 2014 Dec;12(12):2092-103.e1-6. doi: 10.1016/j.cgh.2014.02.024. Epub 2014 Feb 25. PMID: 24582567. |
| 3 | Chalasani N, Vuppalanchi R, Rinella M, Middleton MS, Siddiqui MS, Barritt AS 4th, Kolterman O, Flores O, Alonso C, Iruarrizaga-Lejarreta M, Gil-Redondo R, Sirlin CB, Zemel MB. Randomised clinical trial: a leucine-metformin-sildenafil combination (NS-0200) vs placebo in patients with non-alcoholic fatty liver disease. Aliment Pharmacol Ther. 2018 Jun;47(12):1639-1651. doi: 10.1111/apt.14674. Epub 2018 Apr 25. PMID: 29696666; PMCID: PMC6001629. |
| 4 | Della Corte C, Carpino G, De Vito R, De Stefanis C, Alisi A, Cianfarani S, Overi D, Mosca A, Stronati L, Cucchiara S, Raponi M, Gaudio E, Byrne CD, Nobili V. Docosahexanoic Acid Plus Vitamin D Treatment Improves Features of NAFLD in Children with Serum Vitamin D Deficiency: Results from a Single Centre Trial. PLoS One. 2016 Dec 15;11(12):e0168216. doi: 10.1371/journal.pone.0168216. PMID: 27977757; PMCID: PMC5158039. |
| 5 | Duseja A, Acharya SK, Mehta M, Chhabra S; Shalimar; Rana S, Das A, Dattagupta S, Dhiman RK, Chawla YK. High potency multistrain probiotic improves liver histology in non-alcoholic fatty liver disease (NAFLD): a randomised, double-blind, proof of concept study. BMJ Open Gastroenterol. 2019 Aug 7;6(1):e000315. doi: 10.1136/bmjgast-2019-000315. PMID: 31423319; PMCID: PMC6688701. |
| 6 | Eriksson JW, Lundkvist P, Jansson PA, Johansson L, Kvarnström M, Moris L, Miliotis T, Forsberg GB, Risérus U, Lind L, Oscarsson J. Effects of dapagliflozin and n-3 carboxylic acids on non-alcoholic fatty liver disease in people with type 2 diabetes: a double-blind randomised placebo-controlled study. Diabetologia. 2018 Sep;61(9):1923-1934. doi: 10.1007/s00125-018-4675-2. Epub 2018 Jul 3. PMID: 29971527; PMCID: PMC6096619. |
| 7 | Flint A, Andersen G, Hockings P, Johansson L, Morsing A, Sundby Palle M, Vogl T, Loomba R, Plum-Mörschel L. Randomised clinical trial: semaglutide versus placebo reduced liver steatosis but not liver stiffness in subjects with non-alcoholic fatty liver disease assessed by magnetic resonance imaging. Aliment Pharmacol Ther. 2021 Nov;54(9):1150-1161. doi: 10.1111/apt.16608. Epub 2021 Sep 27. PMID: 34570916; PMCID: PMC9292692. |
| 8 | Fourman LT, Stanley TL, Zheng I, Pan CS, Feldpausch MN, Purdy J, Aepfelbacher J, Buckless C, Tsao A, Corey KE, Chung RT, Torriani M, Kleiner DE, Hadigan CM, Grinspoon SK. Clinical Predictors of Liver Fibrosis Presence and Progression in Human Immunodeficiency Virus-Associated Nonalcoholic Fatty Liver Disease. Clin Infect Dis. 2021 Jun 15;72(12):2087-2094. doi: 10.1093/cid/ciaa382. PMID: 32270862; PMCID: PMC8204775. |
| 9 | Geier A, Eichinger M, Stirnimann G, Semela D, Tay F, Seifert B, Tschopp O, Bantel H, Jahn D, Marques Maggio E, Saleh L, Bischoff-Ferrari HA, Müllhaupt B, Dufour JF. Treatment of non-alcoholic steatohepatitis patients with vitamin D: a double-blinded, randomized, placebo-controlled pilot study. Scand J Gastroenterol. 2018 Sep;53(9):1114-1120. doi: 10.1080/00365521.2018.1501091. Epub 2018 Sep 29. PMID: 30270688. |
| 10 | Guo W, Tian W, Lin L, Xu X. Liraglutide or insulin glargine treatments improves hepatic fat in obese patients with type 2 diabetes and nonalcoholic fatty liver disease in twenty-six weeks: A randomized placebo-controlled trial. Diabetes Res Clin Pract. 2020 Dec;170:108487. doi: 10.1016/j.diabres.2020.108487. Epub 2020 Oct 6. PMID: 33035599. |
| 11 | Gurka MJ, Mack JA, Chi X, DeBoer MD. Use of metabolic syndrome severity to assess treatment with vitamin E and pioglitazone for non-alcoholic steatohepatitis. J Gastroenterol Hepatol. 2021 Jan;36(1):249-256. doi: 10.1111/jgh.15131. Epub 2020 Jul 6. PMID: 32506513; PMCID: PMC7719569. |
| 12 | Harrison SA, Wong VW, Okanoue T, Bzowej N, Vuppalanchi R, Younes Z, Kohli A, Sarin S, Caldwell SH, Alkhouri N, Shiffman ML, Camargo M, Li G, Kersey K, Jia C, Zhu Y, Djedjos CS, Subramanian GM, Myers RP, Gunn N, Sheikh A, Anstee QM, Romero-Gomez M, Trauner M, Goodman Z, Lawitz EJ, Younossi Z; STELLAR-3; STELLAR-4 Investigators. Selonsertib for patients with bridging fibrosis or compensated cirrhosis due to NASH: Results from randomized phase III STELLAR trials. J Hepatol. 2020 Jul;73(1):26-39. doi: 10.1016/j.jhep.2020.02.027. Epub 2020 Mar 6. PMID: 32147362. |
| 13 | Hoofnagle JH, Van Natta ML, Kleiner DE, Clark JM, Kowdley KV, Loomba R, Neuschwander-Tetri BA, Sanyal AJ, Tonascia J; Non-alcoholic Steatohepatitis Clinical Research Network (NASH CRN). Vitamin E and changes in serum alanine aminotransferase levels in patients with non-alcoholic steatohepatitis. Aliment Pharmacol Ther. 2013 Jul;38(2):134-43. doi: 10.1111/apt.12352. Epub 2013 May 29. PMID: 23718573; PMCID: PMC3775262. |
| 14 | Johansen ML, Schou M, Rossignol P, Holm MR, Rasmussen J, Brandt N, Frandsen M, Chabanova E, Dela F, Faber J, Kistorp C. Effect of the mineralocorticoid receptor antagonist eplerenone on liver fat and metabolism in patients with type 2 diabetes: A randomized, double-blind, placebo-controlled trial (MIRAD trial). Diabetes Obes Metab. 2019 Oct;21(10):2305-2314. doi: 10.1111/dom.13809. Epub 2019 Jul 5. PMID: 31183945. |
| 15 | Kazierad DJ, Chidsey K, Somayaji VR, Bergman AJ, Birnbaum MJ, Calle RA. Inhibition of ketohexokinase in adults with NAFLD reduces liver fat and inflammatory markers: A randomized phase 2 trial. Med. 2021 Jul 9;2(7):800-813.e3. doi: 10.1016/j.medj.2021.04.007. Epub 2021 Apr 27. PMID: 35590219. |
| 16 | Kessoku T, Imajo K, Kobayashi T, Honda Y, Kato T, Ogawa Y, Tomeno W, Kato S, Higurashi T, Yoneda M, Kirikoshi H, Kubota K, Taguri M, Yamanaka T, Usuda H, Wada K, Saito S, Nakajima A. Efficacy, safety, and tolerability of lubiprostone for the treatment of non-alcoholic fatty liver disease in adult patients with constipation: The LUBIPRONE, double-blind, randomised, placebo-controlled study design. Contemp Clin Trials. 2018 Jun;69:40-47. doi: 10.1016/j.cct.2018.04.002. Epub 2018 Apr 5. PMID: 29627620. |
| 17 | Lin SC, Ang B, Hernandez C, Bettencourt R, Jain R, Salotti J, Richards L, Kono Y, Bhatt A, Aryafar H, Lin GY, Valasek MA, Sirlin CB, Brouha S, Loomba R. Cardiovascular risk assessment in the treatment of nonalcoholic steatohepatitis: a secondary analysis of the MOZART trial. Therap Adv Gastroenterol. 2016 Mar;9(2):152-61. doi: 10.1177/1756283X15621232. PMID: 26929777; PMCID: PMC4749859. |
| 18 | Loomba R, Kayali Z, Noureddin M, Ruane P, Lawitz EJ, Bennett M, Wang L, Harting E, Tarrant JM, McColgan BJ, Chung C, Ray AS, Subramanian GM, Myers RP, Middleton MS, Lai M, Charlton M, Harrison SA. GS-0976 Reduces Hepatic Steatosis and Fibrosis Markers in Patients With Nonalcoholic Fatty Liver Disease. Gastroenterology. 2018 Nov;155(5):1463-1473.e6. doi: 10.1053/j.gastro.2018.07.027. Epub 2018 Jul 27. PMID: 30059671; PMCID: PMC6318218. |
| 19 | Middleton MS, Van Natta ML, Heba ER, Alazraki A, Trout AT, Masand P, Brunt EM, Kleiner DE, Doo E, Tonascia J, Lavine JE, Shen W, Hamilton G, Schwimmer JB, Sirlin CB; NASH Clinical Research Network. Diagnostic accuracy of magnetic resonance imaging hepatic proton density fat fraction in pediatric nonalcoholic fatty liver disease. Hepatology. 2018 Mar;67(3):858-872. doi: 10.1002/hep.29596. Epub 2018 Jan 26. PMID: 29028128; PMCID: PMC6211296. |
| 20 | Neuschwander-Tetri BA, Loomba R, Sanyal AJ, Lavine JE, Van Natta ML, Abdelmalek MF, Chalasani N, Dasarathy S, Diehl AM, Hameed B, Kowdley KV, McCullough A, Terrault N, Clark JM, Tonascia J, Brunt EM, Kleiner DE, Doo E; NASH Clinical Research Network. Farnesoid X nuclear receptor ligand obeticholic acid for non-cirrhotic, non-alcoholic steatohepatitis (FLINT): a multicentre, randomised, placebo-controlled trial. Lancet. 2015 Mar 14;385(9972):956-65. doi: 10.1016/S0140-6736(14)61933-4. Epub 2014 Nov 7. Erratum in: Lancet. 2015 Mar 14;385(9972):946. Erratum in: Lancet. 2016 Apr 16;387(10028):1618. PMID: 25468160; PMCID: PMC4447192. |
| 21 | Newsome PN, Palmer M, Freilich B, Sheikh MY, Sheikh A, Sarles H, Herring R, Mantry P, Kayali Z, Hassanein T, Lee HM, Aithal GP; Volixibat in Adults study group. Volixibat in adults with non-alcoholic steatohepatitis: 24-week interim analysis from a randomized, phase II study. J Hepatol. 2020 Aug;73(2):231-240. doi: 10.1016/j.jhep.2020.03.024. Epub 2020 Mar 29. PMID: 32234329. |
| 22 | Nobili V, Manco M, Devito R, Di Ciommo V, Comparcola D, Sartorelli MR, Piemonte F, Marcellini M, Angulo P. Lifestyle intervention and antioxidant therapy in children with nonalcoholic fatty liver disease: a randomized, controlled trial. Hepatology. 2008 Jul;48(1):119-28. doi: 10.1002/hep.22336. PMID: 18537181. |
| 23 | Oscarsson J, Önnerhag K, Risérus U, Sundén M, Johansson L, Jansson PA, Moris L, Nilsson PM, Eriksson JW, Lind L. Effects of free omega-3 carboxylic acids and fenofibrate on liver fat content in patients with hypertriglyceridemia and non-alcoholic fatty liver disease: A double-blind, randomized, placebo-controlled study. J Clin Lipidol. 2018 Nov-Dec;12(6):1390-1403.e4. doi: 10.1016/j.jacl.2018.08.003. Epub 2018 Aug 10. PMID: 30197273. |
| 24 | Parker HM, Cohn JS, O'Connor HT, Garg ML, Caterson ID, George J, Johnson NA. Effect of Fish Oil Supplementation on Hepatic and Visceral Fat in Overweight Men: A Randomized Controlled Trial. Nutrients. 2019 Feb 23;11(2):475. doi: 10.3390/nu11020475. PMID: 30813440; PMCID: PMC6413081. |
| 25 | Sanyal AJ, Abdelmalek MF, Suzuki A, Cummings OW, Chojkier M; EPE-A Study Group. No significant effects of ethyl-eicosapentanoic acid on histologic features of nonalcoholic steatohepatitis in a phase 2 trial. Gastroenterology. 2014 Aug;147(2):377-84.e1. doi: 10.1053/j.gastro.2014.04.046. Epub 2014 May 9. PMID: 24818764. |
| 26 | Scherer T, Wolf P, Smajis S, Gaggini M, Hackl M, Gastaldelli A, Klimek P, Einwallner E, Marculescu R, Luger A, Fürnsinn C, Trattnig S, Buettner C, Krššák M, Krebs M. Chronic Intranasal Insulin Does Not Affect Hepatic Lipids but Lowers Circulating BCAAs in Healthy Male Subjects. J Clin Endocrinol Metab. 2017 Apr 1;102(4):1325-1332. doi: 10.1210/jc.2016-3623. PMID: 28323986; PMCID: PMC6283450. |
| 27 | Scorletti E, Bhatia L, McCormick KG, Clough GF, Nash K, Hodson L, Moyses HE, Calder PC, Byrne CD; WELCOME Study. Effects of purified eicosapentaenoic and docosahexaenoic acids in nonalcoholic fatty liver disease: results from the Welcome* study. Hepatology. 2014 Oct;60(4):1211-21. doi: 10.1002/hep.27289. PMID: 25043514. |
| 28 | Stanley TL, Fourman LT, Zheng I, McClure CM, Feldpausch MN, Torriani M, Corey KE, Chung RT, Lee H, Kleiner DE, Hadigan CM, Grinspoon SK. Relationship of IGF-1 and IGF-Binding Proteins to Disease Severity and Glycemia in Nonalcoholic Fatty Liver Disease. J Clin Endocrinol Metab. 2021 Jan 23;106(2):e520-e533. doi: 10.1210/clinem/dgaa792. PMID: 33125080; PMCID: PMC7823253. |
| 29 | Sun Y, Chen G, Chen S, Wang Y, Hu Y, Zhao Y. Efficacy and safety of Jian-Pi Huo-Xue granule for non-alcoholic fatty liver disease: study protocol for a randomized, double-blind, placebo-controlled trial. Trials. 2022 Jun 2;23(1):455. doi: 10.1186/s13063-022-06393-8. PMID: 35655225; PMCID: PMC9164345. |
| 30 | Van Wagner LB, Koppe SW, Brunt EM, Gottstein J, Gardikiotes K, Green RM, Rinella ME. Pentoxifylline for the treatment of non-alcoholic steatohepatitis: a randomized controlled trial. Ann Hepatol. 2011 Jul-Sep;10(3):277-86. PMID: 21677329. |
| 31 | Wah Kheong C, Nik Mustapha NR, Mahadeva S. A Randomized Trial of Silymarin for the Treatment of Nonalcoholic Steatohepatitis. Clin Gastroenterol Hepatol. 2017 Dec;15(12):1940-1949.e8. doi: 10.1016/j.cgh.2017.04.016. Epub 2017 Apr 15. PMID: 28419855. |
| 32 | Harrison SA, Ruane PJ, Freilich B, Neff G, Patil R, Behling C, Hu C, Shringarpure R, de Temple B, Fong E, Tillman EJ, Rolph T, Cheng A, Yale K. A randomized, double-blind, placebo-controlled phase IIa trial of efruxifermin for patients with compensated NASH cirrhosis. JHEP Rep. 2022 Aug 23;5(1):100563. doi: 10.1016/j.jhepr.2022.100563. PMID: 36644237; PMCID: PMC9832280. |
| 33 | Loomba R, Abdelmalek MF, Armstrong MJ, Jara M, Kjær MS, Krarup N, Lawitz E, Ratziu V, Sanyal AJ, Schattenberg JM, Newsome PN; NN9931-4492 investigators. Semaglutide 2·4 mg once weekly in patients with non-alcoholic steatohepatitis-related cirrhosis: a randomised, placebo-controlled phase 2 trial. Lancet Gastroenterol Hepatol. 2023 Jun;8(6):511-522. doi: 10.1016/S2468-1253(23)00068-7. Epub 2023 Mar 16. PMID: 36934740; PMCID: PMC10792518. |
| 34 | Barcelos STA, Silva-Sperb AS, Moraes HA, Longo L, de Moura BC, Michalczuk MT, Uribe-Cruz C, Cerski CTS, da Silveira TR, Dall'Alba V, Álvares-da-Silva MR. Oral 24-week probiotics supplementation did not decrease cardiovascular risk markers in patients with biopsy proven NASH: A double-blind placebo-controlled randomized study. Ann Hepatol. 2023 Jan-Feb;28(1):100769. doi: 10.1016/j.aohep.2022.100769. Epub 2022 Oct 8. PMID: 36216309. |
| 35 | Vos MB, Van Natta ML, Blondet NM, Dasarathy S, Fishbein M, Hertel P, Jain AK, Karpen SJ, Lavine JE, Mohammad S, Miriel LA, Molleston JP, Mouzaki M, Sanyal A, Sharkey EP, Schwimmer JB, Tonascia J, Wilson LA, Xanthakos SA; NASH Clinical Research Network. Randomized placebo-controlled trial of losartan for pediatric NAFLD. Hepatology. 2022 Aug;76(2):429-444. doi: 10.1002/hep.32403. Epub 2022 Feb 28. PMID: 35133671; PMCID: PMC9288975. |
| 36 | Grobbee EJ, de Jong VD, Schrieks IC, Tushuizen ME, Holleboom AG, Tardif JC, Lincoff AM, Schwartz GG, Castro Cabezas M, Grobbee DE. Improvement of non-invasive tests of liver steatosis and fibrosis as indicators for non-alcoholic fatty liver disease in type 2 diabetes mellitus patients with elevated cardiovascular risk profile using the PPAR-α/γ agonist aleglitazar. PLoS One. 2022 Nov 15;17(11):e0277706. doi: 10.1371/journal.pone.0277706. PMID: 36378671; PMCID: PMC9665379. |
| 37 | Ratziu V, Harrison SA, Loustaud-Ratti V, Bureau C, Lawitz E, Abdelmalek M, Alkhouri N, Francque S, Girma H, Darteil R, Couchoux H, Wolf M, Sanyal A, Vonderscher J, Scalfaro P. Hepatic and renal improvements with FXR agonist vonafexor in individuals with suspected fibrotic NASH. J Hepatol. 2023 Mar;78(3):479-492. doi: 10.1016/j.jhep.2022.10.023. Epub 2022 Nov 9. PMID: 36334688. |
| 38 | Nogueira MA, Oliveira CP, Ferreira Alves VA, Stefano JT, Rodrigues LS, Torrinhas RS, Cogliati B, Barbeiro H, Carrilho FJ, Waitzberg DL. Omega-3 polyunsaturated fatty acids in treating non-alcoholic steatohepatitis: A randomized, double-blind, placebo-controlled trial. Clin Nutr. 2016 Jun;35(3):578-86. doi: 10.1016/j.clnu.2015.05.001. Epub 2015 May 21. PMID: 26047766. |
| 39 | Argo CK, Patrie JT, Lackner C, Henry TD, de Lange EE, Weltman AL, Shah NL, Al-Osaimi AM, Pramoonjago P, Jayakumar S, Binder LP, Simmons-Egolf WD, Burks SG, Bao Y, Taylor AG, Rodriguez J, Caldwell SH. Effects of n-3 fish oil on metabolic and histological parameters in NASH: a double-blind, randomized, placebo-controlled trial. J Hepatol. 2015 Jan;62(1):190-7. doi: 10.1016/j.jhep.2014.08.036. Epub 2014 Sep 6. PMID: 25195547; PMCID: PMC4272639. |
| 40 | Gastaldelli A, Harrison S, Belfort-Aguiar R, Hardies J, Balas B, Schenker S, Cusi K. Pioglitazone in the treatment of NASH: the role of adiponectin. Aliment Pharmacol Ther. 2010 Sep;32(6):769-75. doi: 10.1111/j.1365-2036.2010.04405.x. PMID: 20662773. |
| 41 | Gastaldelli A, Sabatini S, Carli F, Gaggini M, Bril F, Belfort-DeAguiar R, Positano V, Barb D, Kadiyala S, Harrison S, Cusi K. PPAR-γ-induced changes in visceral fat and adiponectin levels are associated with improvement of steatohepatitis in patients with NASH. Liver Int. 2021 Nov;41(11):2659-2670. doi: 10.1111/liv.15005. Epub 2021 Jul 21. PMID: 34219361; PMCID: PMC9290929. |
| 42 | Bhatt DL, Bays HE, Miller M, Cain JE 3rd, Wasilewska K, Andrawis NS, Parli T, Feng S, Sterling L, Tseng L, Hartsfield CL, Agollah GD, Mansbach H, Kastelein JJP; ENTRIGUE Principal Investigators. The FGF21 analog pegozafermin in severe hypertriglyceridemia: a randomized phase 2 trial. Nat Med. 2023 Jul;29(7):1782-1792. doi: 10.1038/s41591-023-02427-z. Epub 2023 Jun 24. Erratum in: Nat Med. 2024 Mar 4;: PMID: 37355760; PMCID: PMC10353930. |
| 43 | Dufour JF, Oneta CM, Gonvers JJ, Bihl F, Cerny A, Cereda JM, Zala JF, Helbling B, Steuerwald M, Zimmermann A; Swiss Association for the Study of the Liver. Randomized placebo-controlled trial of ursodeoxycholic acid with vitamin e in nonalcoholic steatohepatitis. Clin Gastroenterol Hepatol. 2006 Dec;4(12):1537-43. doi: 10.1016/j.cgh.2006.09.025. PMID: 17162245. |
| 44 | Amin NB et al. Efficacy and safety of an orally administered DGAT2 inhibitor alone or coadministered with a liver-targeted ACC inhibitor in adults with non-alcoholic steatohepatitis (NASH): rationale and design of the phase II, dose-ranging, dose-finding, randomised, placebo-controlled MIRNA (Metabolic Interventions to Resolve NASH with fibrosis) study. BMJ Open. 2022 Mar 30;12(3):e056159. doi: 10.1136/bmjopen-2021-056159. PMID: 35354614; PMCID: PMC8968568. |

## 15. Publication bias analysis


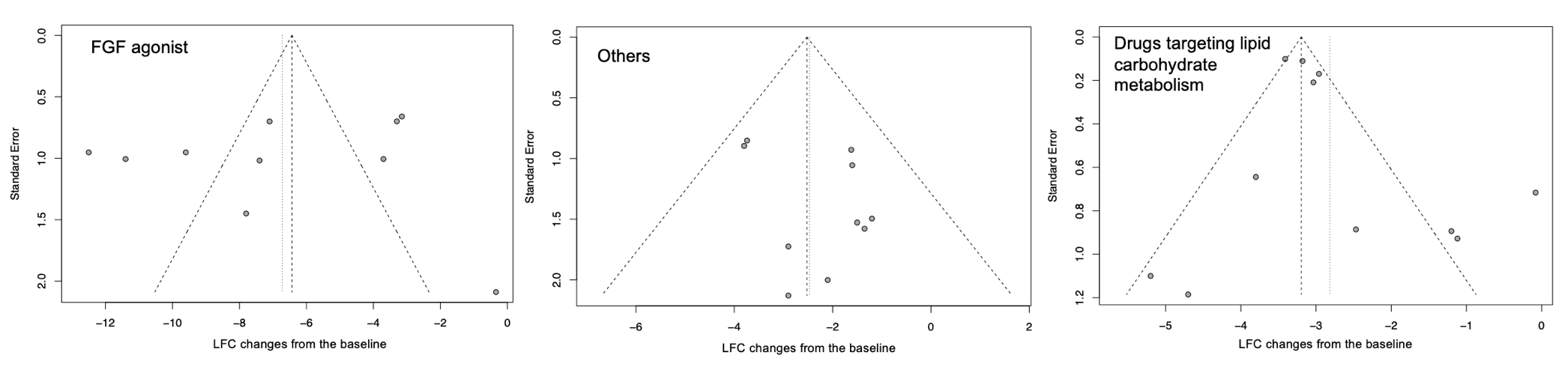


Figure S15: Funnel plot of meta-analysis for ΔΔLFC across different drug classes

The horizontal axis represents effect size, while the vertical axis represents standard error. This figure includes only those studies that reported standard error (SE) values. Due to the number of reported SE values were less than 10 for FXR agonists, Drugs targeting cell death, inflammation or fibrosis and PPAR agonists, these were not included in the analysis. The graph indicates that drugs such as FGF agonists and those affecting glucose and lipid metabolism exhibit high heterogeneity, making it difficult to determine the presence of publication bias. Drugs targeting cellular inflammation, general inflammation, fibrosis, and PPAR agonists have too few data points to assess for publication bias. Apart from these, other drug classes show low publication bias, with data points evenly distributed around the center.

## 16. Comparison of observed data and model predictions in the Phase III clinical trial of Resmetirom


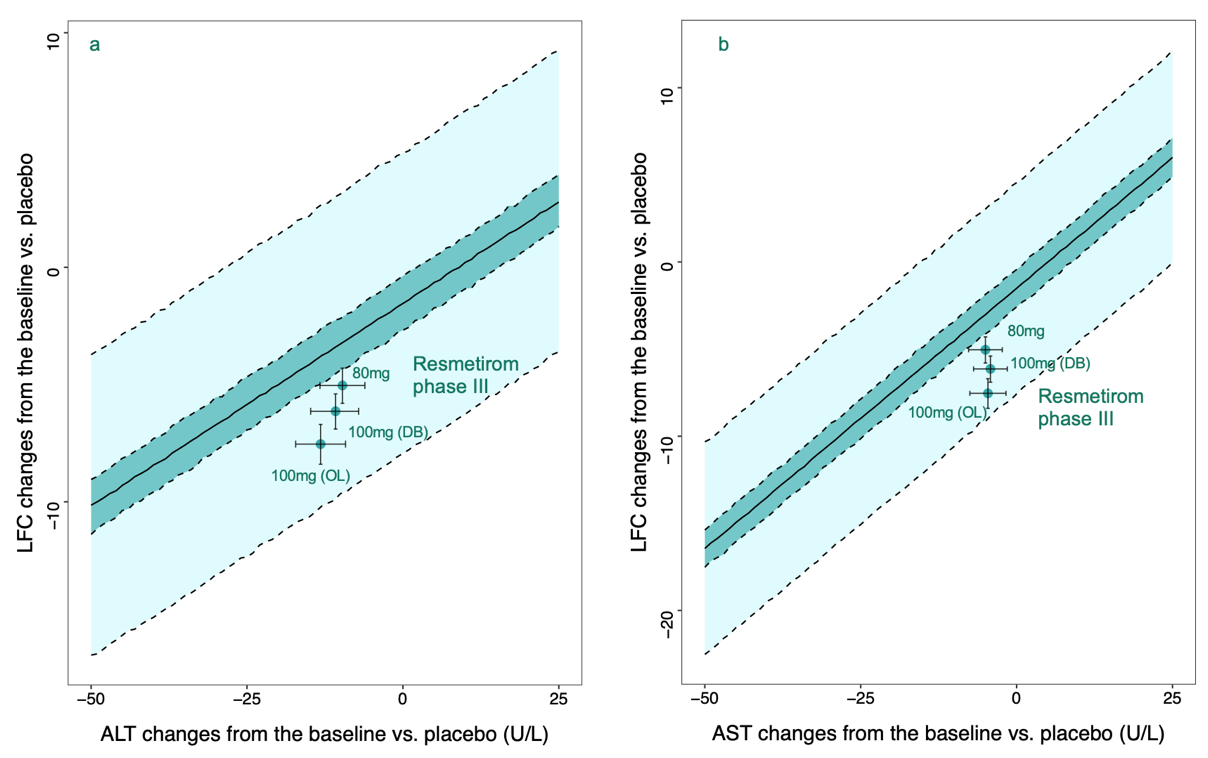


Figure S16. Comparison of observed data and predicted results from ALT model (a) and AST model (b) in the Phase III clinical trial of Resmetirom. Points represent observed values of ΔΔLFC along with corresponding ΔΔALT and ΔΔAST from the Phase III trial of Resmetirom, with error bars indicating the standard errors. Light shaded areas denote the 95% CIs for ΔΔLFC predicted by the ALT or AST models, while dark shaded areas represent the 95% CIs for the median predicted values. OL: Open label, DB: Double blind
